# Supplementary material for: A multi-regional human brain atlas of chromatin accessibility and gene expression facilitates promoter-isoform resolution genetic fine-mapping
Source: Nat Commun. 2024 Nov 22;15:10113. doi: 10.1038/s41467-024-54448-y (PMC11584674; doi:10.1038/s41467-024-54448-y)
Supplement: Supplementary file 1 — Supplementary Information [file 41467_2024_54448_MOESM1_ESM.docx]

Supplementary information for

# A Multi-Regional Human Brain Atlas of Chromatin Accessibility and Gene Expression Facilitates Promoter-Isoform Resolution Genetic Fine-Mapping

#

## Supplementary Figures


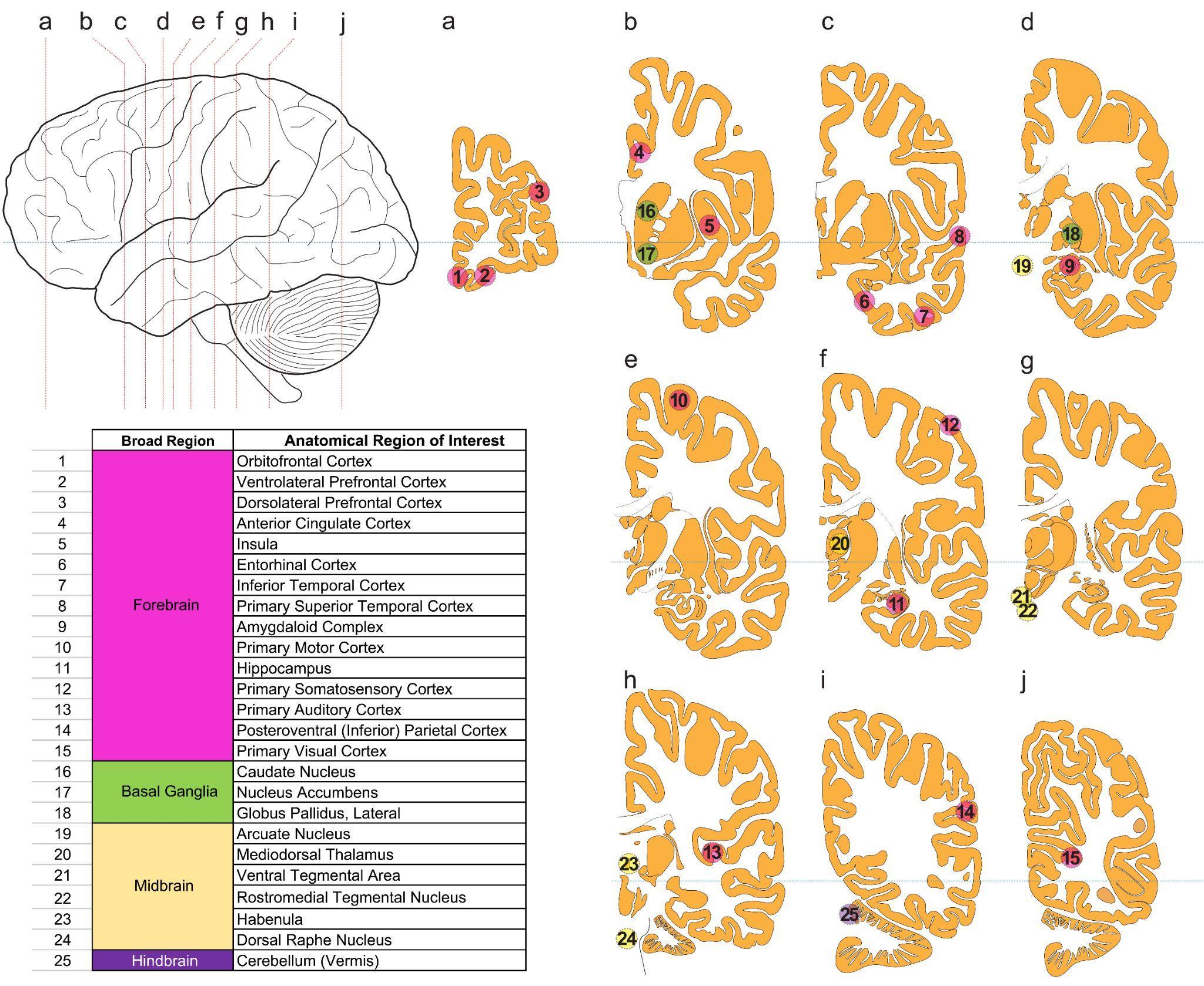


###### Supplementary Fig. 1 | Anatomical localization of the brain regions assayed in the study. Tissue samples used in this study were isolated from 25 functionally distinct brain regions from 6 control individuals.


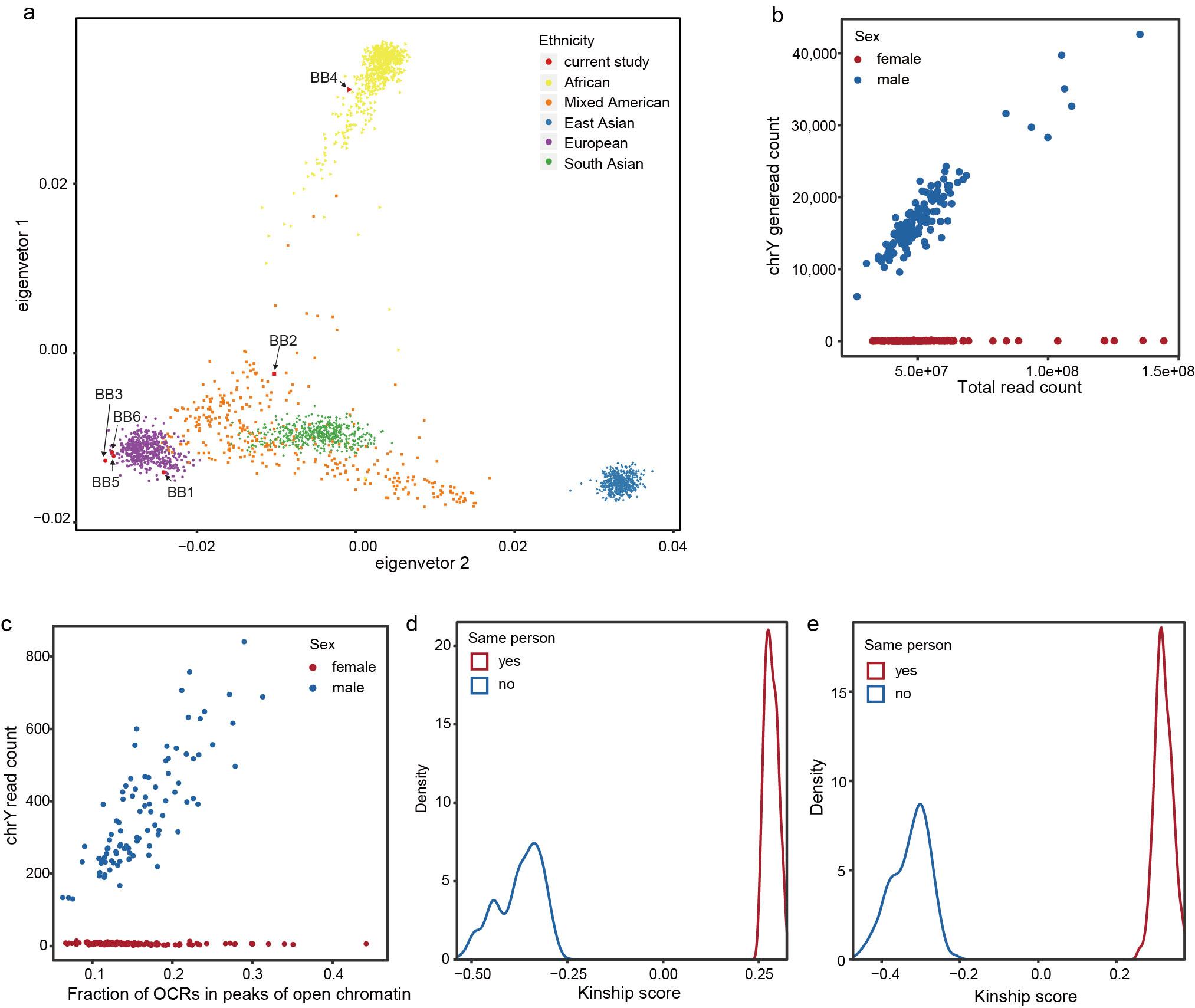


###### Supplementary Fig. 2 | Genetic and sex concordance of the samples. a, Principal component analysis of five main 1KG population clusters with individuals from the current study. b, RNA-seq sex check based on measuring the number of reads mapped on genes located on chromosome Y (genes located on pseudoautosomal regions are not counted). c, ATAC-seq sex check based on measuring the number of reads mapped on chromosome Y. d, Genotype concordance (as calculated by KING) based on pair-wise comparison of genotypes called from RNA-seq samples with genotypes from whole-genome sequencing. The histogram shows that none of the non-matching pairs have a positive KING value which indicates full genotype concordance. e, Genotype concordance (as calculated by KING) based on pair-wise comparison of genotypes called from ATAC-seq samples with genotypes from whole-genome sequencing. The histogram shows that none of the non-matching pairs have a positive KING value which indicates full genotype concordance. Source data are provided as a Source Data file.

######


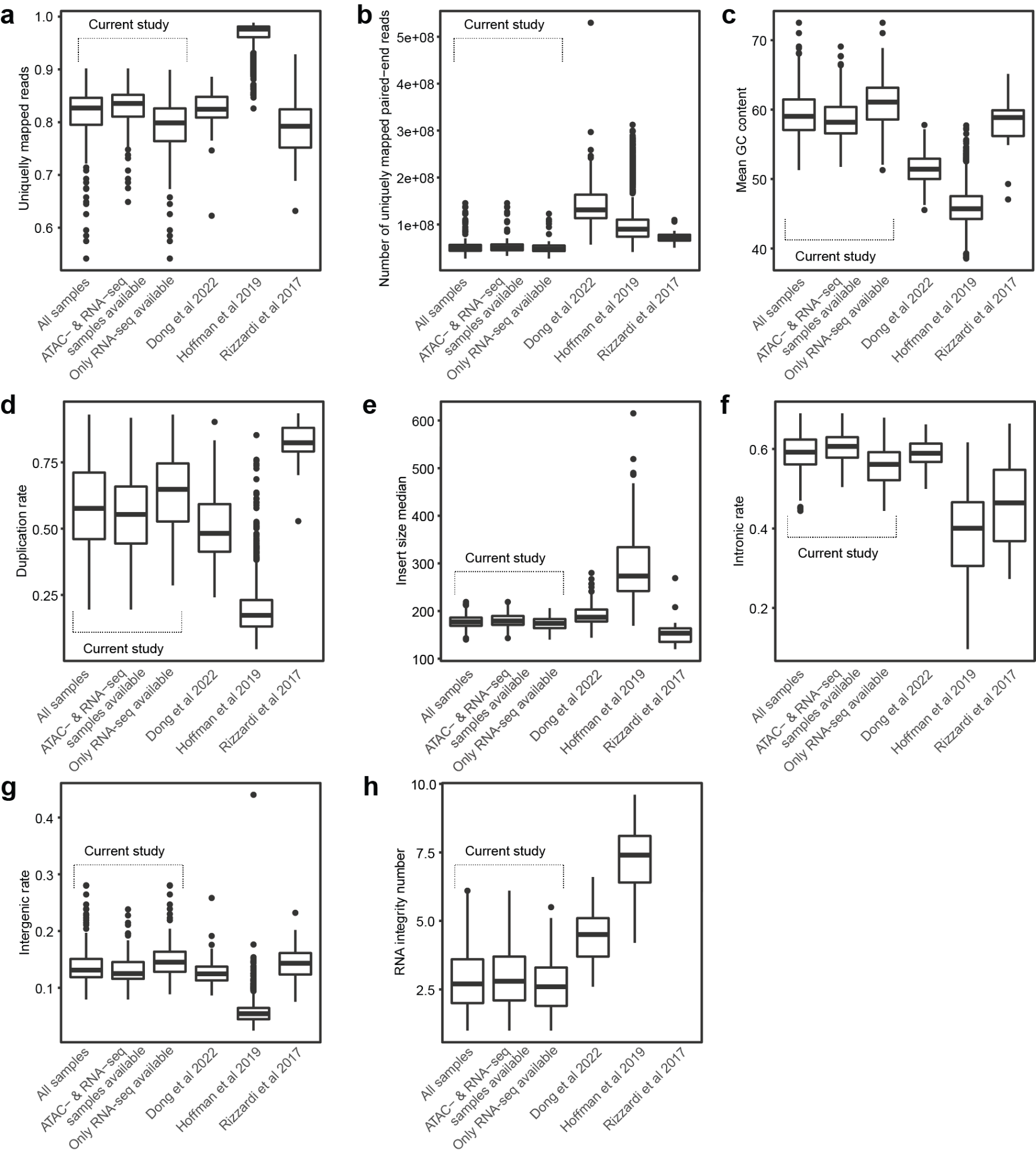


###### Supplementary Fig. 3 | Comparison of the RNA-seq Quality Control Metrics with published results. Boxplots showing the quality control metrics between our results (all samples, ATAC- & RNA-seq samples available, only RNA-seq available samples), and published data sets (Dong et al., 2022[^1^](https://sciwheel.com/work/citation?ids=13672697&pre=&suf=&sa=0), Hoffman et al. 2019 [^2^](https://sciwheel.com/work/citation?ids=8218833&pre=&suf=&sa=0), Rizzardi et al., 2017[^3^](https://sciwheel.com/work/citation?ids=6272553&pre=&suf=&sa=0) ) generated using postmortem human brains by similar assay, i.e. FANS RNA-seq or homogenate RNA-seq. a, Fraction of uniquely mapped reads by STAR aligner. b, Number of uniquely mapped pair-end reads. c, Mean GC content. d, Ratio of short fragments (50-100bp) to long fragments (150-200bp) used for detecting under- or over-transposition. e, Median insert size between paired reads. f, Intronic rate. g, Intergenic rate. h, RNA integrity number. Source data are provided as a Source Data file.


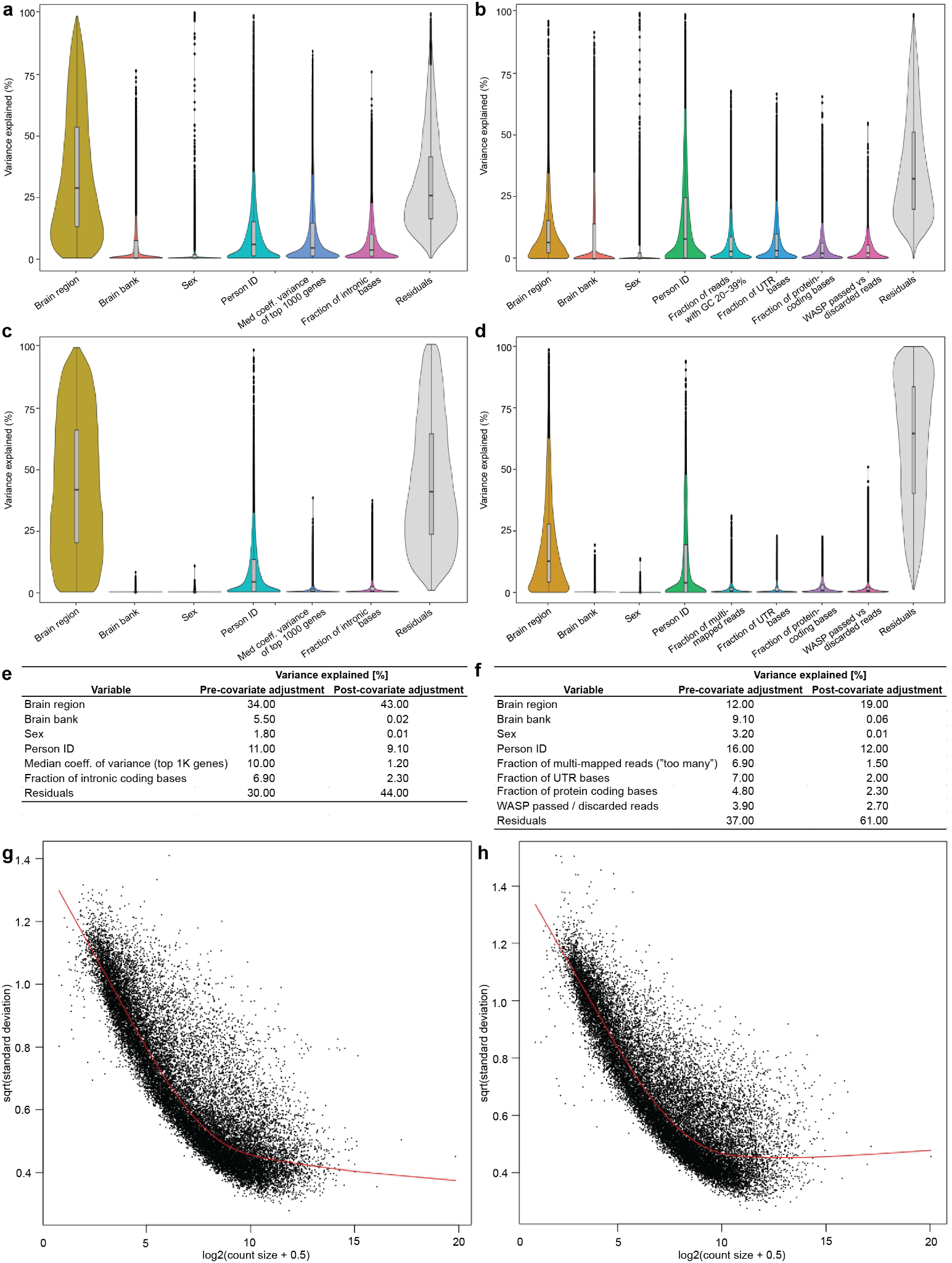


###### Supplementary Fig. 4 | Covariate selection and statistical modeling for RNA-seq analysis. a-d, Violin plots of the percentage of variance explained by each covariate plus residuals over all the neuronal (a, c) and non-neuronal (b, d) samples, before (a-b) and after (c-d) covariate adjustment. e-f, Tabular overview of variance before and after adjustment for covariates for neuronal (e) and non-neuronal (f) samples. g-h, Peak-wise means and variances of neuronal (g) and non-neuronal (h) samples after adjustments for covariates. Peaks are represented by black points with LOWESS trends shown in red. Source data are provided as a Source Data file.

######


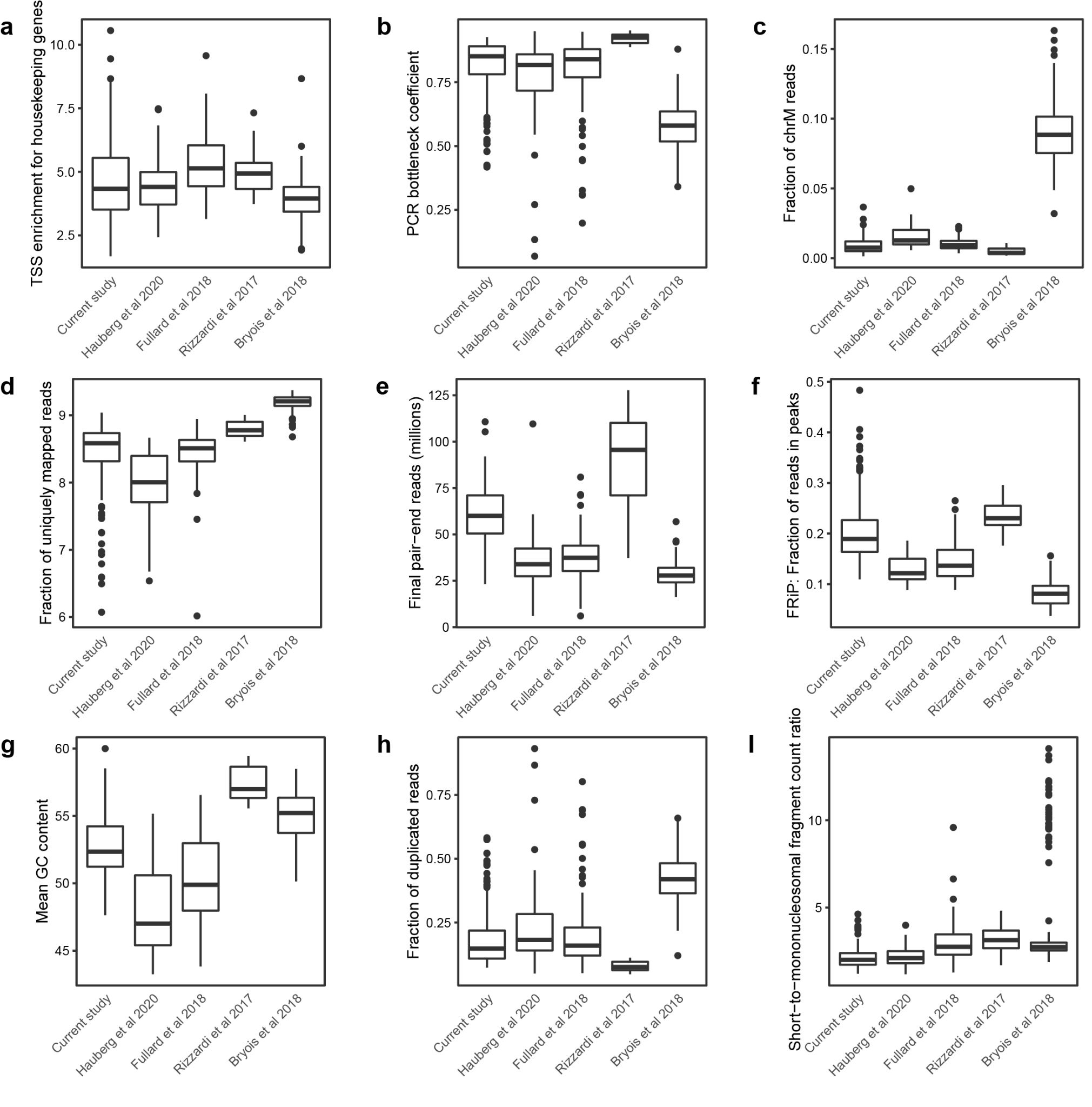


###### Supplementary Fig. 5 | Comparison of ATAC-seq Quality Control Metrics with published results. Boxplots showing the quality control metrics between our results (current study), and published data sets (Hauberg et al., 2020[^4^](https://sciwheel.com/work/citation?ids=9969657&pre=&suf=&sa=0), Fullard et al. 2018[^5^](https://sciwheel.com/work/citation?ids=5486493&pre=&suf=&sa=0), Rizzardi et al., 2017[^3^](https://sciwheel.com/work/citation?ids=6272553&pre=&suf=&sa=0)) generated on postmortem human brains by a similar assay, i.e. FANS ATAC-seq or homogenate ATAC-seq. a, TSS enrichment for housekeeping genes. b, PCR bottleneck coefficient reflecting the complexity of the library (ENCODE quality metrics, https://www.encodeproject.org/data-standards/atac-seq/atac-encode4/). c, Fraction of chrM reads; note that the higher values for Bryois et al. are caused by their use of whole tissue and not sorted nuclei. d, Fraction of uniquely mapped reads by STAR aligner. e, Number of “final” paired-end reads, i.e. after filtering non-uniquely mapped reads, duplicated reads and chrM reads. f, Fraction of reads in consensus peaksets. Note that each study has a different consensus peakset. g, Mean GC content. h, Fraction of duplicated reads. i, Ratio of short fragments (50-100bp) to long fragments (150-200bp) used for detecting under- or over-transposition. Source data are provided as a Source Data file.


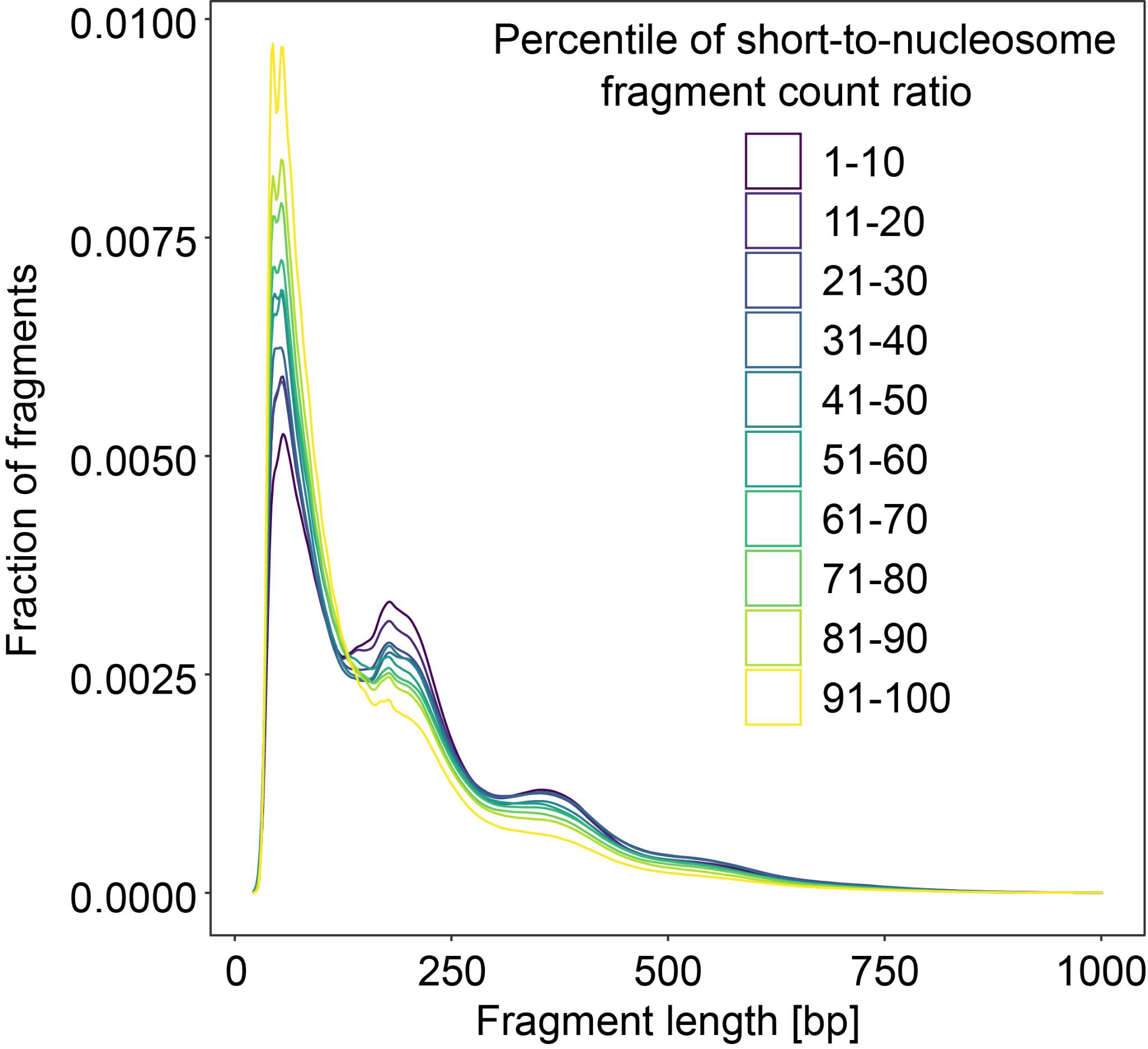


###### Supplementary Fig. 6 | Fragment length distribution of the ATAC-seq data. Averaged fragment length distribution. Samples were assigned to ten bins, from the lowest to the largest short-to-nucleosome fragment count ratio (calculated as the ratio of short fragments (50-100bp) to long fragments (150-200bp)). Source data are provided as a Source Data file.


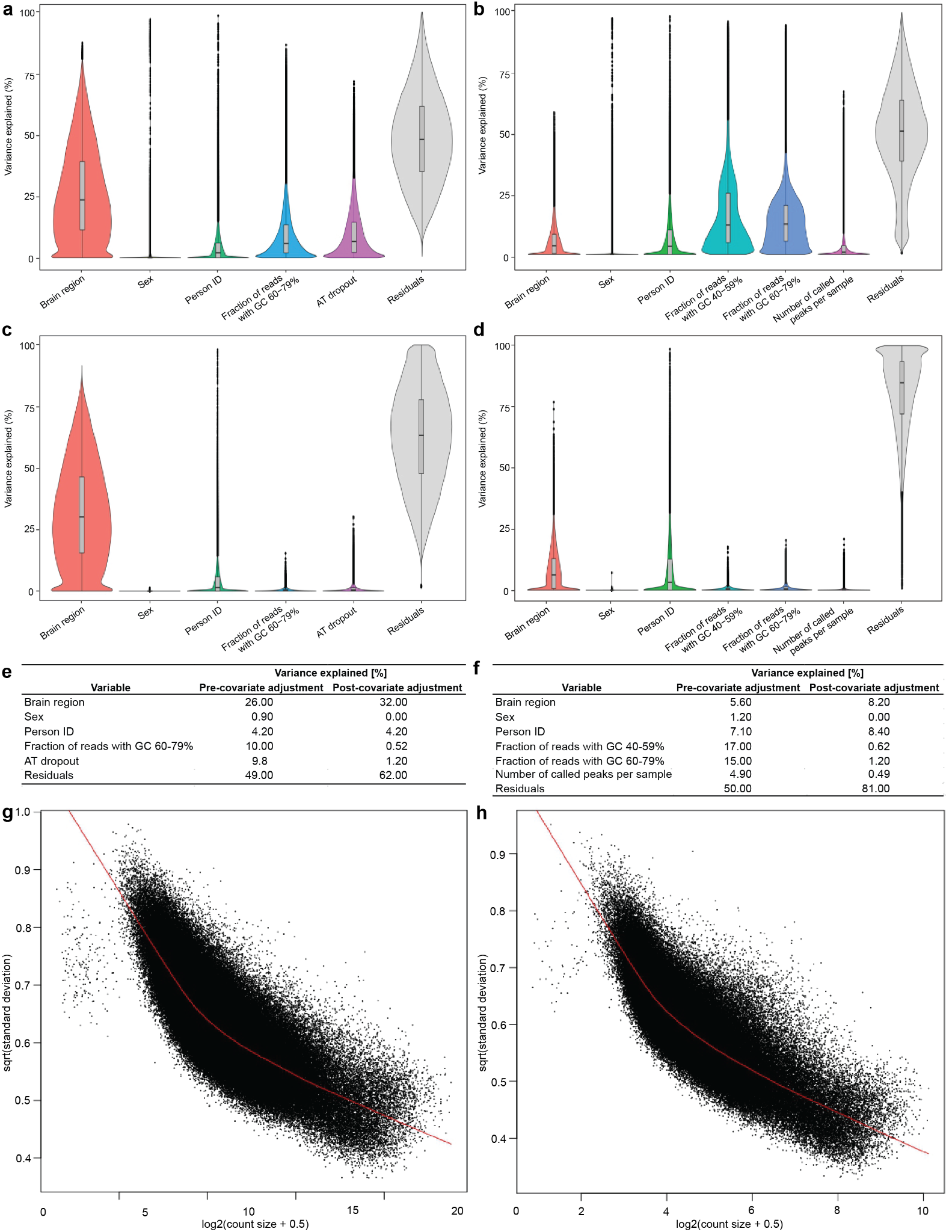


###### Supplementary Fig. 7 | Covariate selection and statistical modeling for ATAC-seq analysis. a-d, Violin plots of the percentage of variance explained by each covariate plus residuals over all neuronal (a, c) and non-neuronal (b, d) samples, before (a-b) and after (c-d) covariate adjustment. e-f, Tabular overview of variance before and after adjustment for covariates for neuronal (e) and non-neuronal (f) samples. g-h, Peak-wise means and variances of neuronal (g) and non-neuronal (h) samples after covariate adjustment. Peaks are represented by black points with LOWESS trends shown in red. Source data are provided as a Source Data file.


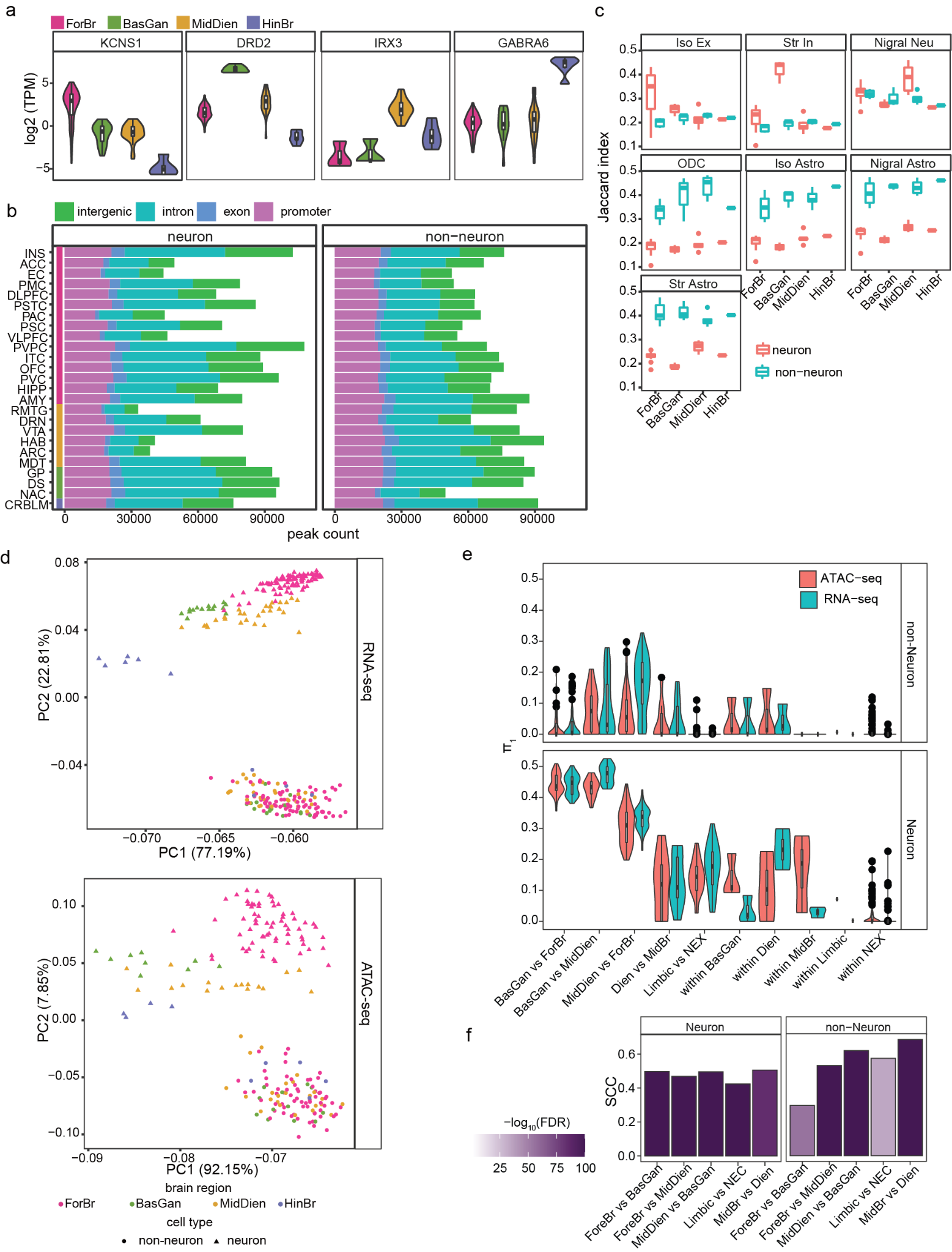


###### Supplementary Fig. 8 | Transcription and chromatin accessibility map across 25 brain regions. a, Neuronal gene expression distribution (log_2_TPM) for the brain region-specific marker genes. Box plot indicates the median, interquartile range (IQR), and 1.5 × IQR. b, The number of peaks and the genomic context profiles merged from the neuronal broad brain regions around. The promoter is defined as areas within 3kb of TSS. c, Jaccard index between our ATAC-seq peaks and a cross-brain region single cell reference[^6^](https://sciwheel.com/work/citation?ids=9909252&pre=&suf=&sa=0), including Isocortical excitatory(ISoEx), Striatal inhibitory (StrIn), Nigral neurons (NigralNeu), ODC (Oligodendrocytes), Isocortical astrocytes (IsoAstro), Nigral astrocytes (NigraLAstro), Striatal astrocytes (StrAstro). d, Clustering of the individual samples for ATAC-seq (N=202) and RNA-seq (N=265) using Principal component analysis. The value within the parenthesis indicates the percentage of variance explained. e, Pairwise statistical dissimilarity (quantified based on the proportion of true tests, π1) across different brain region comparisons. f, Spearman correlation coefficient (SCC) of the log2 fold change between the gene expression and promoter chromatin accessibility across different comparisons (only the genes or OCRs were significant were considered). Source data are provided as a Source Data file.


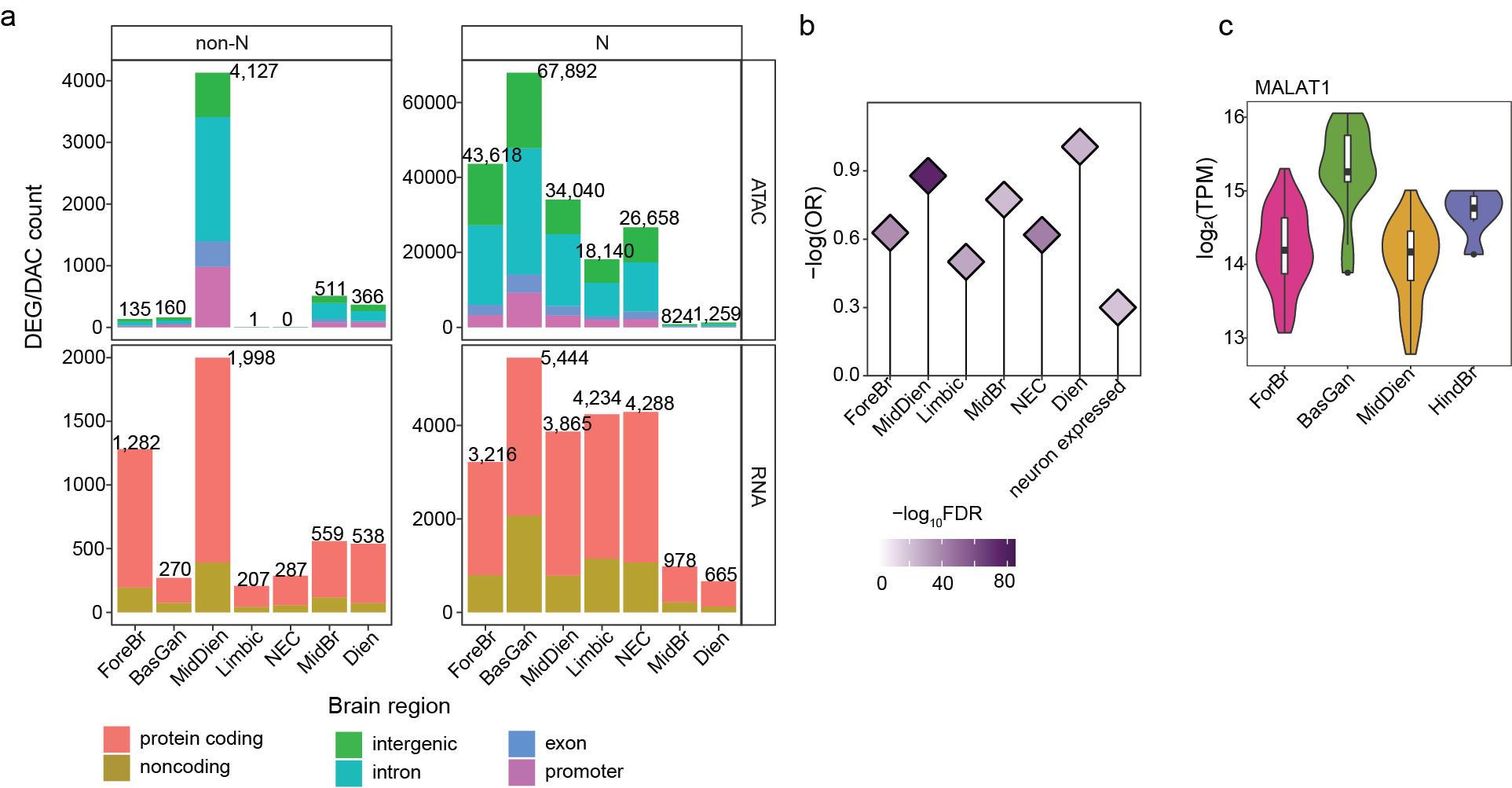


###### Supplementary Fig. 9 | Differential molecular features across broad brain regions. a, Number of differentially expressed genes (DEG) and differentially accessibility chromatin (DAC) for neuronal (N) and non-neuronal (non-N) cells. For broad brain regions, OCRs/genes were considered specific only if they were significantly more accessible/expressed in all pairwise comparisons against the remaining brain regions. For within broad brain region comparisons (Limbic vs NEC, and MidBr vs Dien), only a single pairwise comparison is performed. b, Fisher exact test to examine ncRNA fraction differences between neuronal BasGan DEGs and other DEGs. The heights of the bars represent the enrichment (log odds ratio, OR), while the color indicates the significance (FDR, Benjamini & Hochberg correction, with all FDR values < 10^-16^). c, gene expression (log_2_TPM) of lincRNA MALAT1 in neurons across brain regions. Box plot indicates the median, IQR, and 1.5 × IQR. Source data is provided as a Source Data file.

######

######


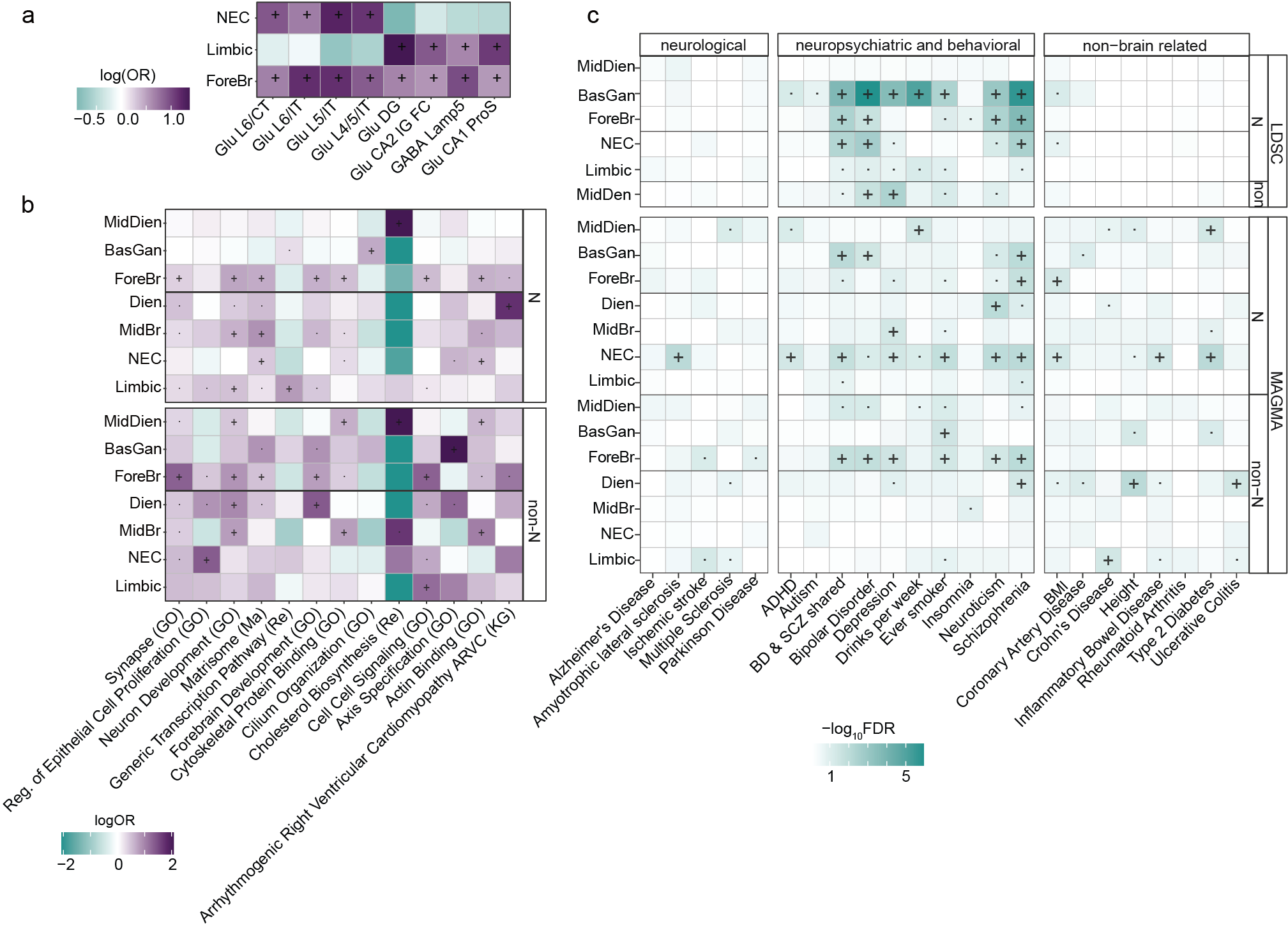


###### Supplementary Fig. 10 | Cell type, pathway, and genetic risk variants associated with broad brain-specific molecular features. a, Cell type enrichment of ForeBr neuronal DEGs with reference from Allen Brain Institute[^7^](https://sciwheel.com/work/citation?ids=11050514&pre=&suf=&sa=0). Glu, glutamatergic neuron. GABA, gabaergic neuron. IT, intratelencephalic neuron. CT, corticothalamic. DG, dentate gyrus. FC, fasciola cinereal. IG, induseum griseum. ProS, prosubiculum. b, biological pathway enrichment for the DEGs. non-N represents non-neurons. The color of the heatmap demonstrates the enrichment odds ratio (OR). One-sided fisher-test significance. ”·”: Nominally significant (*P* < 0.05); ”+”: significant after FDR (Benjamini & Hochberg) correction (FDR < 0.05). non-N denotes non-neurons. c, The significance of stratified LD score regression(upper panel) of DAC, and MAGMA (bottom panel) of DEG across different classes of traits for neurons (N) and non-Neurons (non-N).”·”: Nominally significant (*P* < 0.05); ”+”: significant after FDR correction (FDR < 0.05). Source data is provided as a Source Data file.


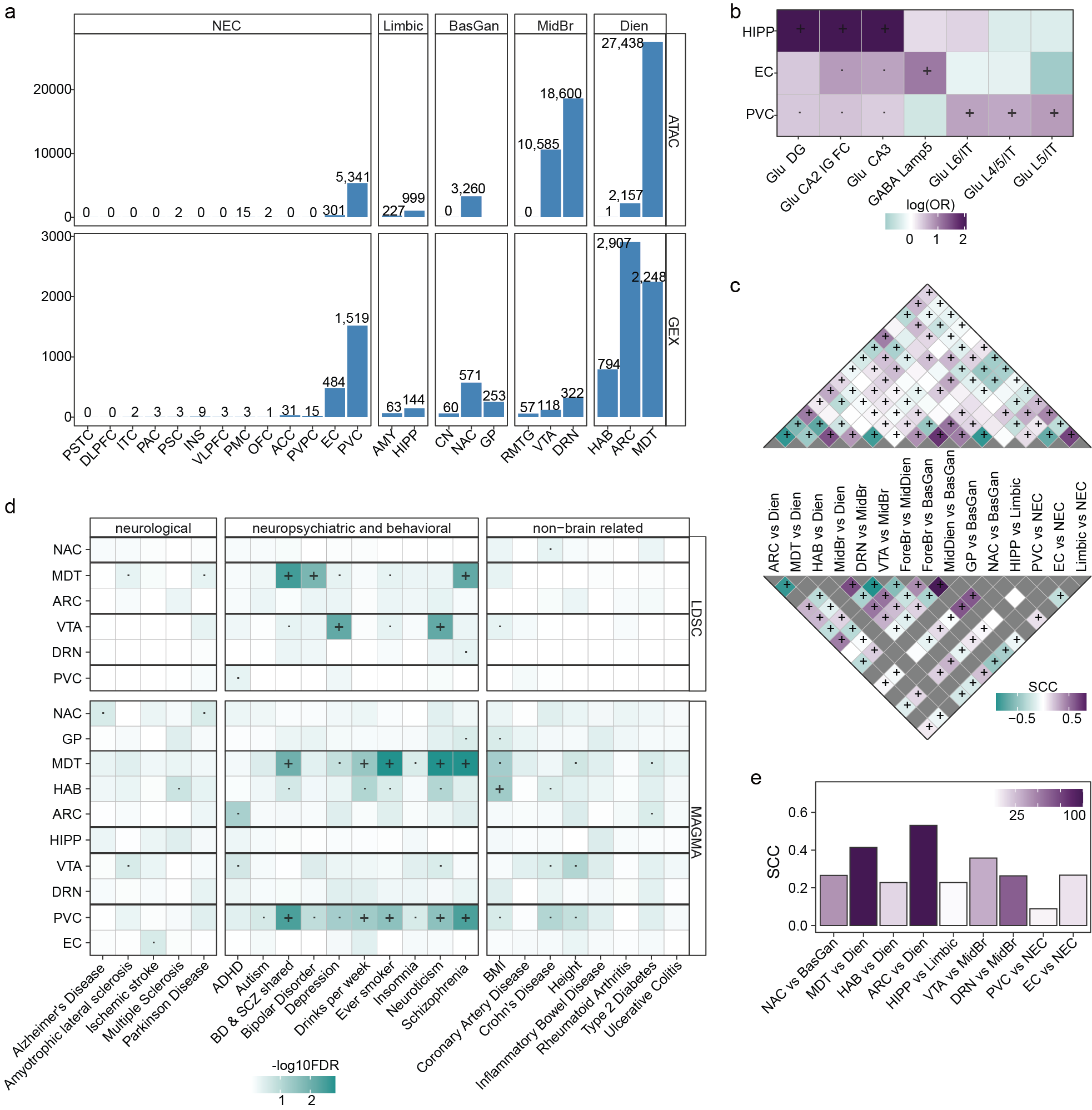


###### Supplementary Fig. 11 | Molecular changes across fine brain regions. a, The number of DEGs and DACs for the fine brain regions in neurons. b, ForeBr neuronal fine brain region DEG enrichment with reference from Allen Brain Institute[^7^](https://sciwheel.com/work/citation?ids=11050514&pre=&suf=&sa=0). Glu, glutamatergic neuron. GABA, gabaergic neuron. IT, intratelencephalic neuron. DG, dentate gyrus. c, spearman correlation between the pairwise comparisons. Only genes that are significant in at least one of the two comparisons were used. ”+”: significant after FDR (Benjamini & Hochberg) correction (FDR < 0.05). d, The upper panel displays the significance of stratified LD score regression for DAC, while the bottom panel shows the significance of MAGMA analysis for DEGs across different classes of traits. ”·”: Nominally significant (*P* < 0.05); ”+”: significant after FDR (Benjamini & Hochberg) correction (FDR < 0.05). e, Spearman correlation coefficient (SCC) of the log2 fold change between the gene expression and promoter chromatin accessibility across different comparisons (only those genes or OCRs that were significant were considered). Source data is provided as a Source Data file.


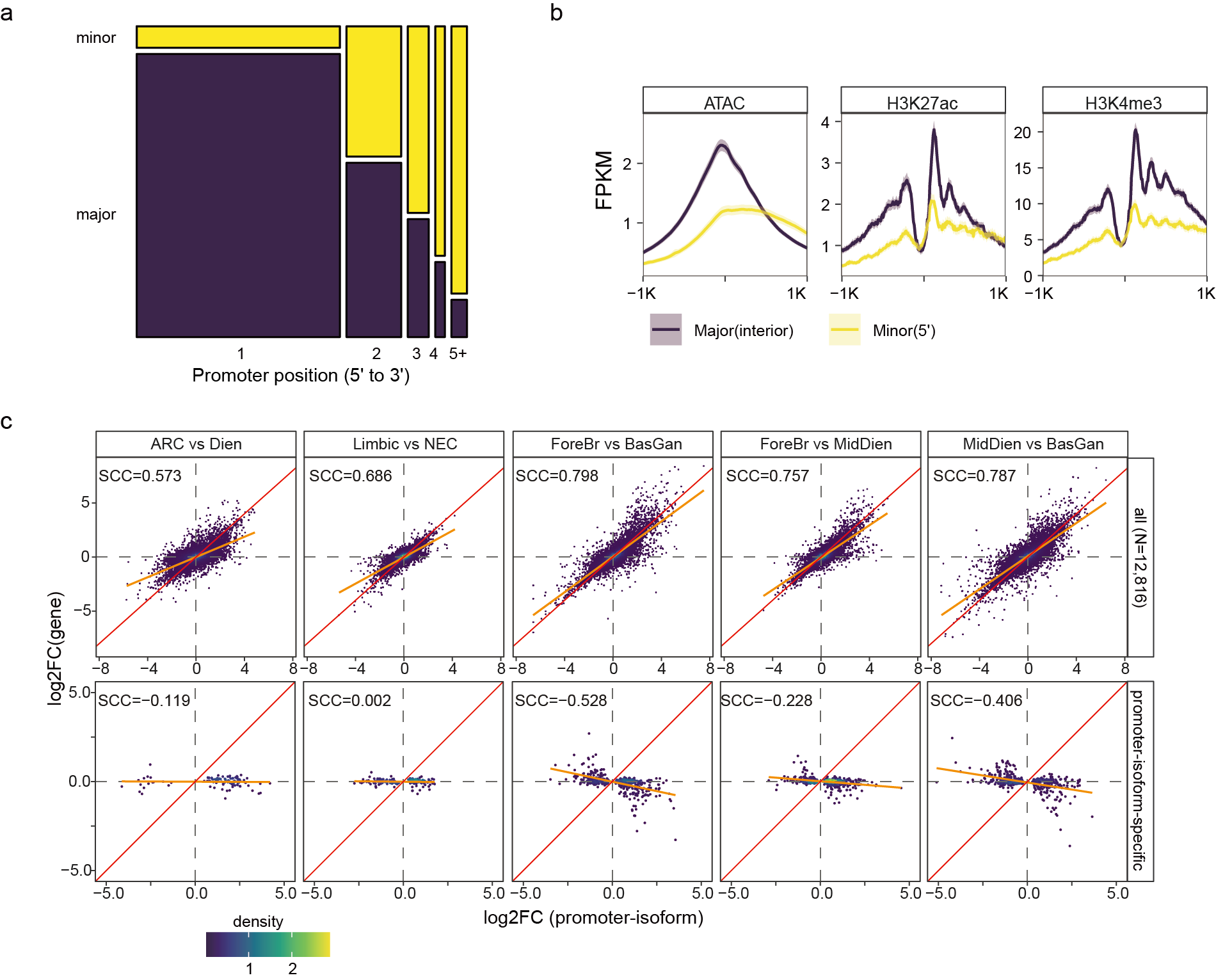


###### Supplementary Fig. 12 | Alternative promoter-isoforms. a, Major/minor promoter proportions across TSSs ranked by position (5’ to 3’, 1 indicating the most 5’), based on multi-promoter genes with at least one active promoter. b, Epigenomic profiles around major interior promoters and non-major 5’ promoters. The shadow shows the 95% confidence intervals.ChIP-seq profiles derived from Dong et al 2022[^1^](https://sciwheel.com/work/citation?ids=13672697&pre=&suf=&sa=0).^.^ c, Spearman correlation coefficient (SCC) between the log2 fold change of gene and promoter-isoform expressions of all promoter-isoforms (upper panel) and non-concordant promoter-isoforms (bottom panel). Source data is provided as a Source Data file.

######
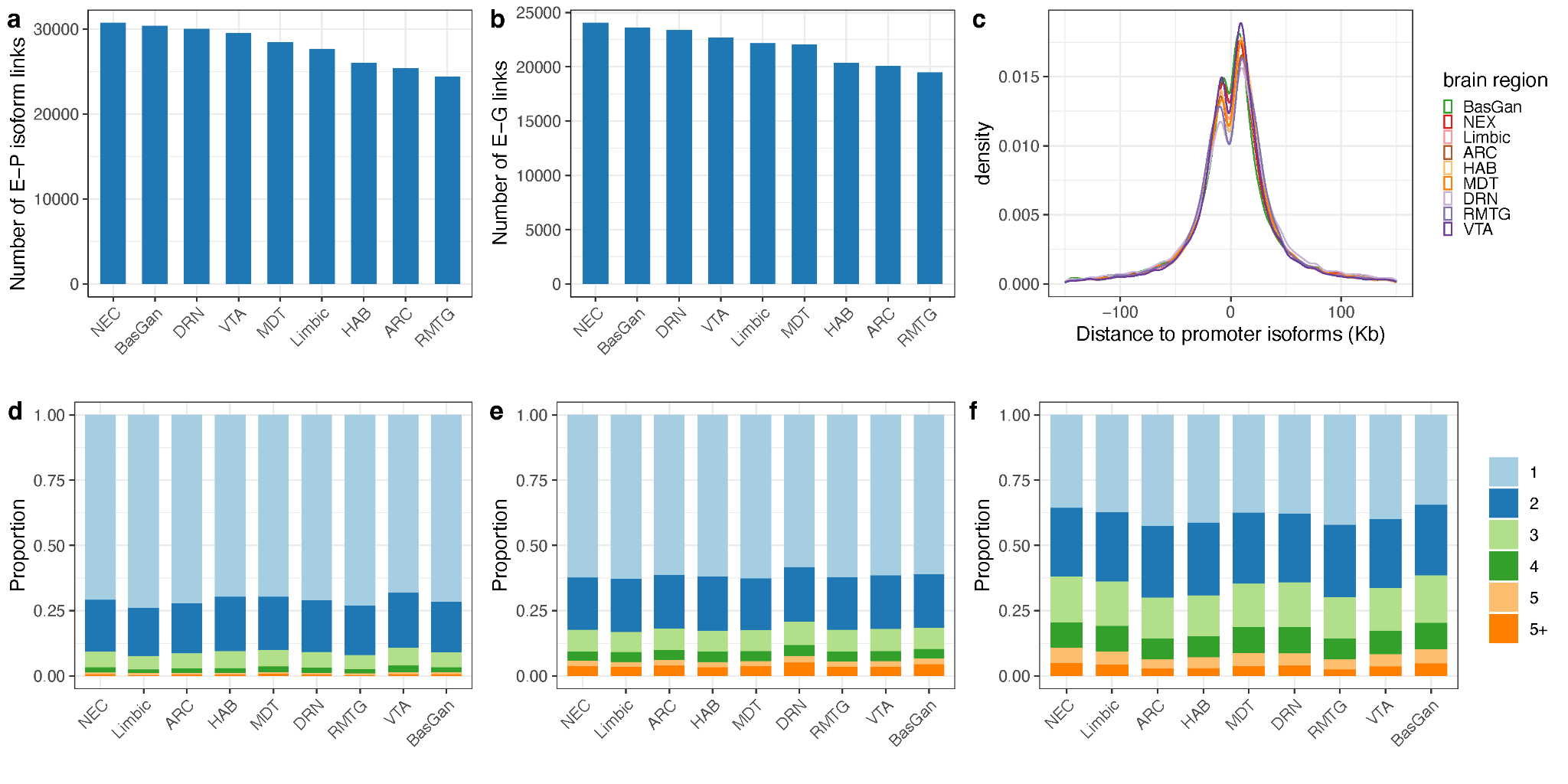
 Supplementary Fig. 13 | The global architecture of E-P isoform links across brain regions. a, The number of E-P links at promoter-isoform resolution. b, The number of enhancer-gene links c, Distribution of the distance between enhancer and target promoter. d, Barplot represents the distribution of the number of E-P links ( promoter-isoform resolution) per gene. e, The distribution of the number of linked isoforms per enhancer. f, The distribution of the number of linked enhancers per gene. Source data is provided as a Source Data file.

######
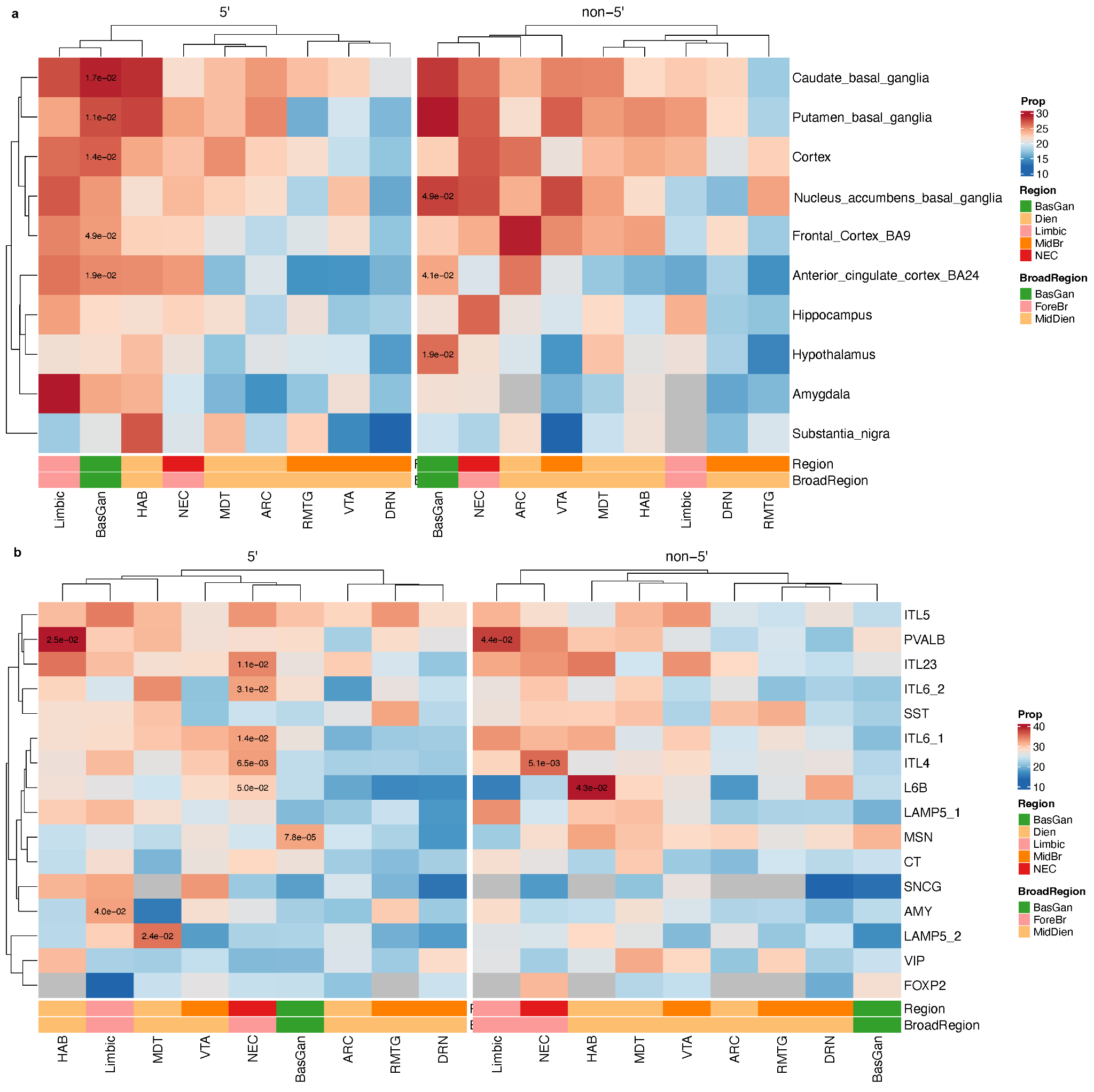


###### Supplementary Fig. **14** | **Validation of** region-unique **E-P links.** Brian region-specific datasets from **a,** the GTEx *cis*-eQTL[^8^](https://sciwheel.com/work/citation?ids=9635829&pre=&suf=&sa=0), and **b,** gene-enhancer coordination from a multi-brain region single cell atlas[^9^](https://sciwheel.com/work/citation?ids=15501918&pre=&suf=&sa=0). The heatmap color represents the proportion of overlapped E-P links, Proportion = (N _validation_ / N_overlap in one region_), see Methods. *P* values were determined using a one-tailed Fisher's exact test to assess whether the validated proportion for one region is higher than that of other regions. Source data is provided as a Source Data file.

#
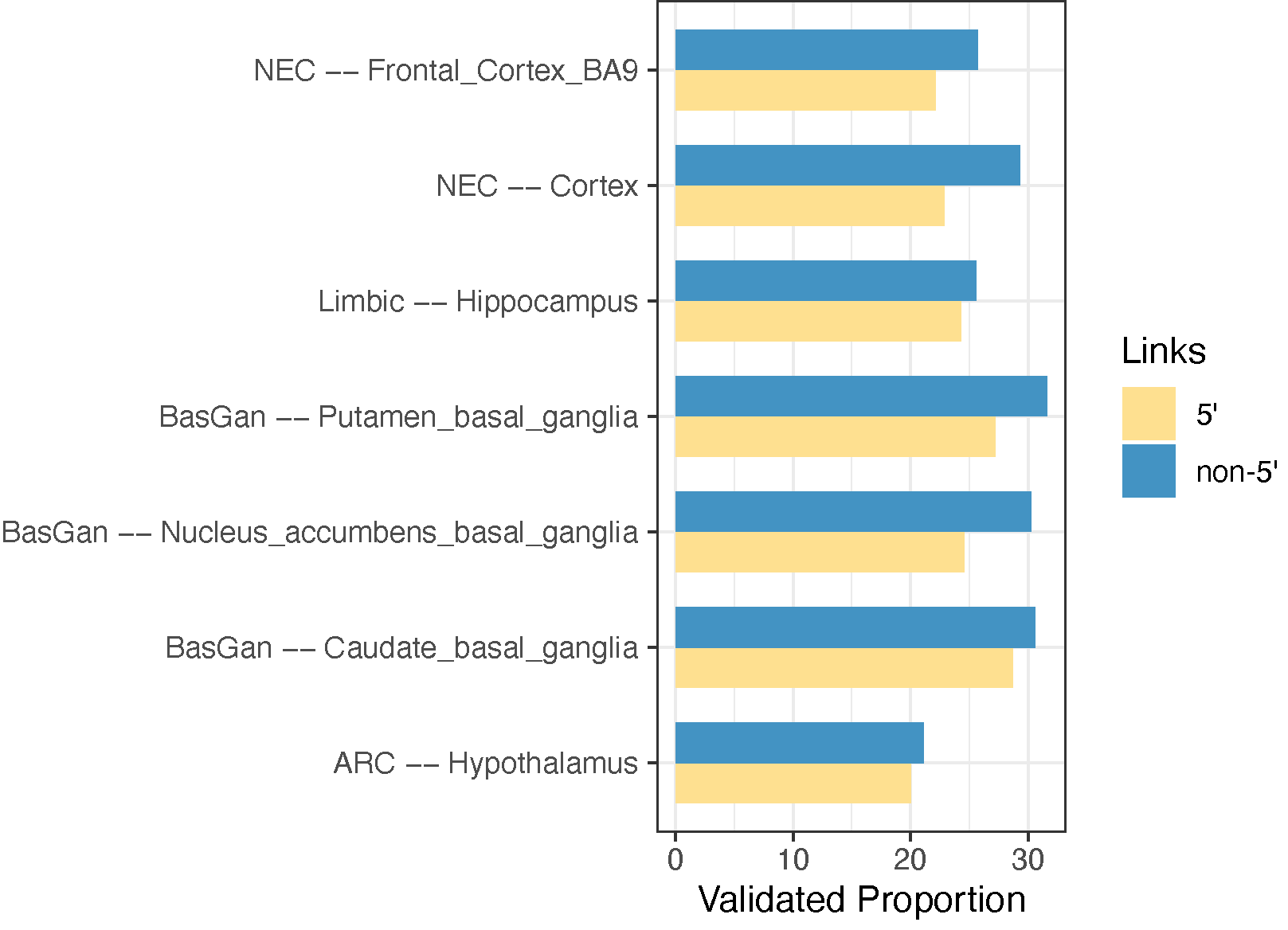


#

###### Supplementary Fig. 15 | The proportion of 5’ and non-5’ links can be validated for matched regions with GTEx eQTL (v8). Source data is provided as a Source Data file.

#
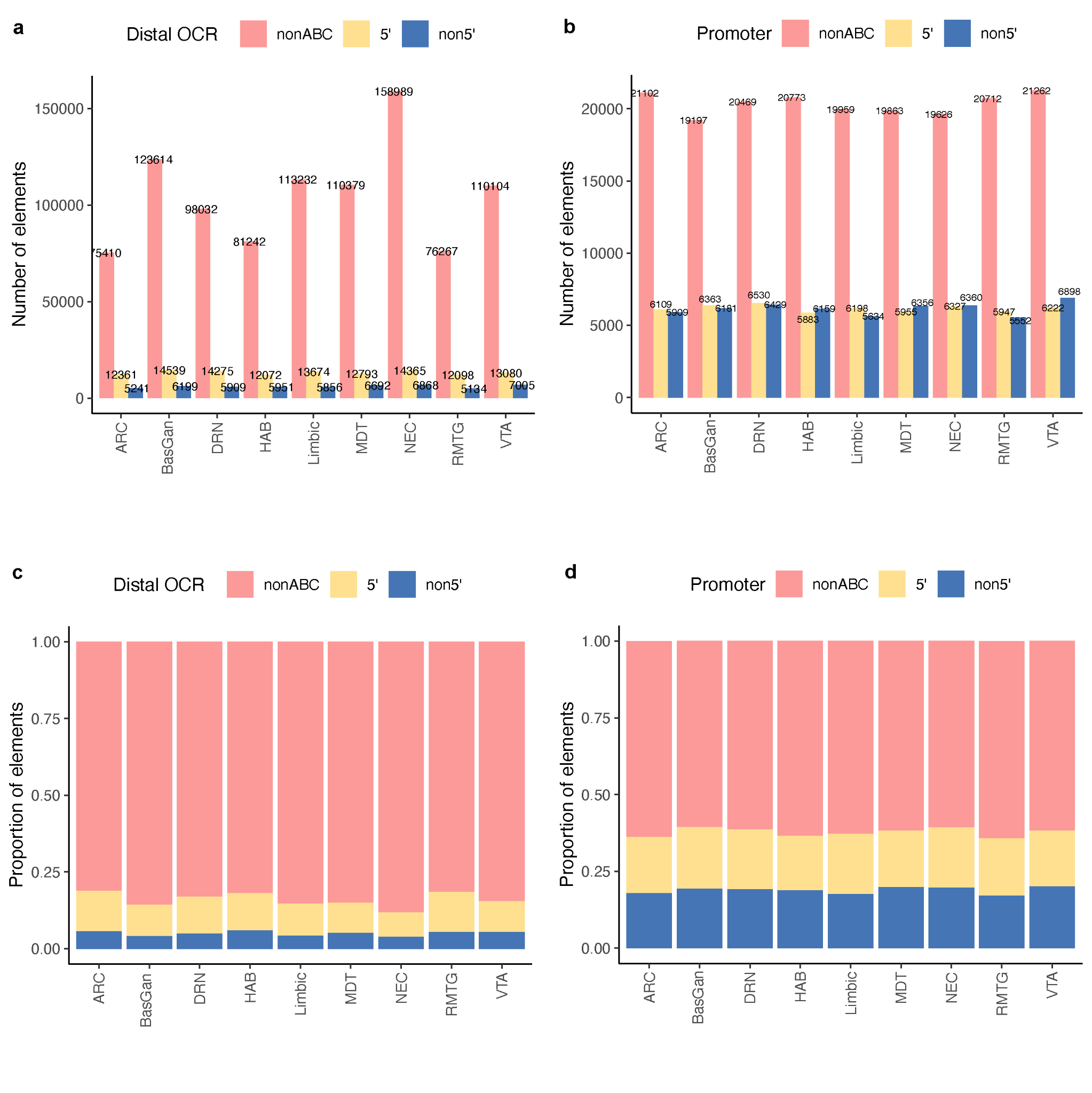


###### Supplementary Fig. 16 | The number and proportion of 5’, non5’, and non-ABC cis-regulatory elements (CREs). a,c, The number (a) and proportion (c) of 5’, non5’, and nonABC distal OCRs identified in each brain region. b,d, The number (b) and proportion (d) of 5’, non5’, and nonABC promoters identified in each brain region.

######
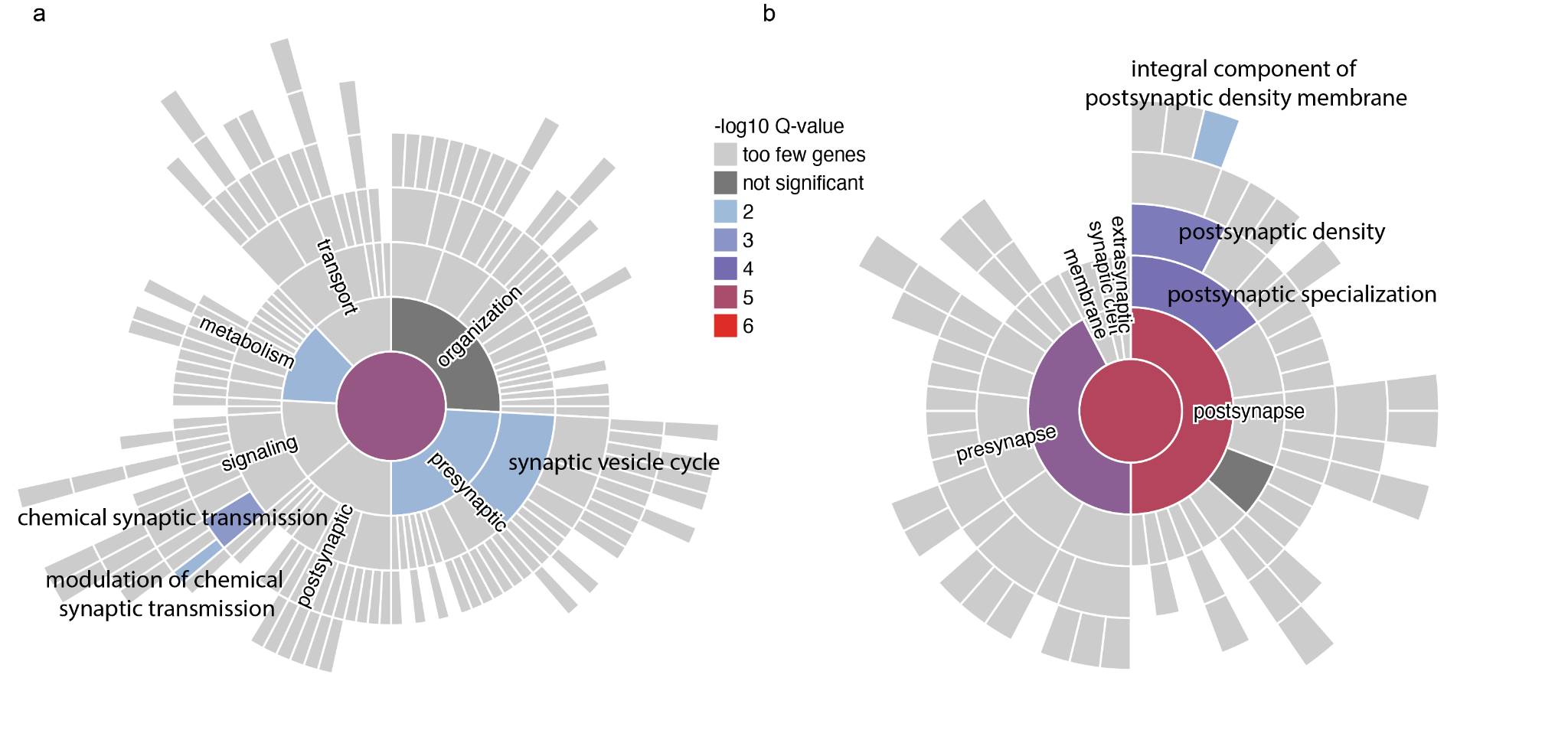
Supplementary Fig. 17 |Synapse function and gene enrichment of SCZ prioritized genes within the SynGO database. a, Enriched cellular components of SCZ prioritized genes. b, Enriched biological processes of SCZ prioritized genes[^10^](https://sciwheel.com/work/citation?ids=7028478&pre=&suf=&sa=0).


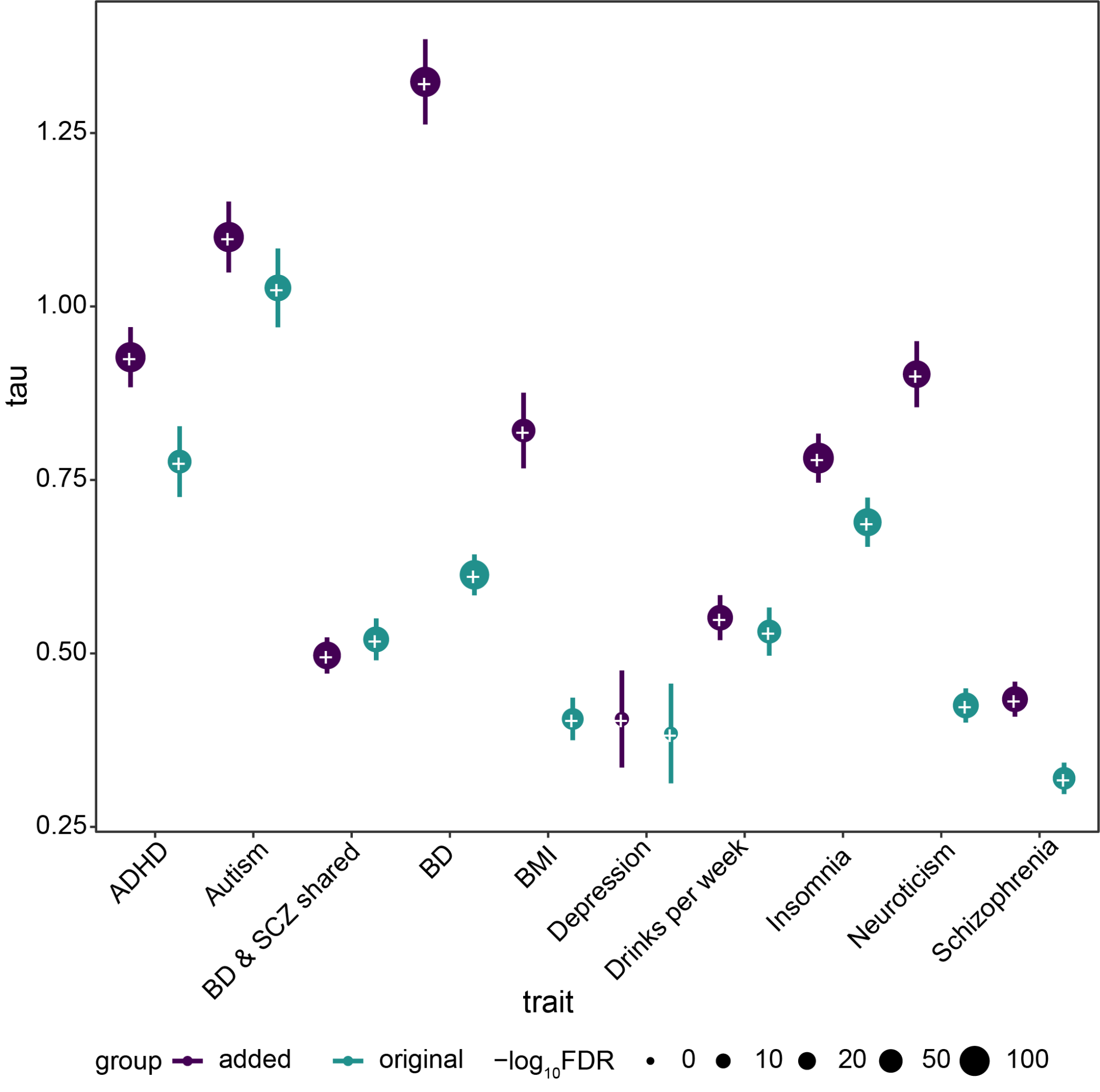


###### Supplementary Fig. 18. | The enrichment of common risk variants in PoPs prioritized genes. The enrichment of common variants for different neuropsychiatric traits for genes with the top 10% PoPS score that imputed with the original feature and updated features (added). The y-axis represents the per SNP heritability (tau ± s.e. ), and the size represents the FDR value. ”·”: Nominally significant (p<0.05); ”+”: significant after FDR (Benjamini & Hochberg) correction (FDR < 0.05). Source data is provided as a Source Data file.

######
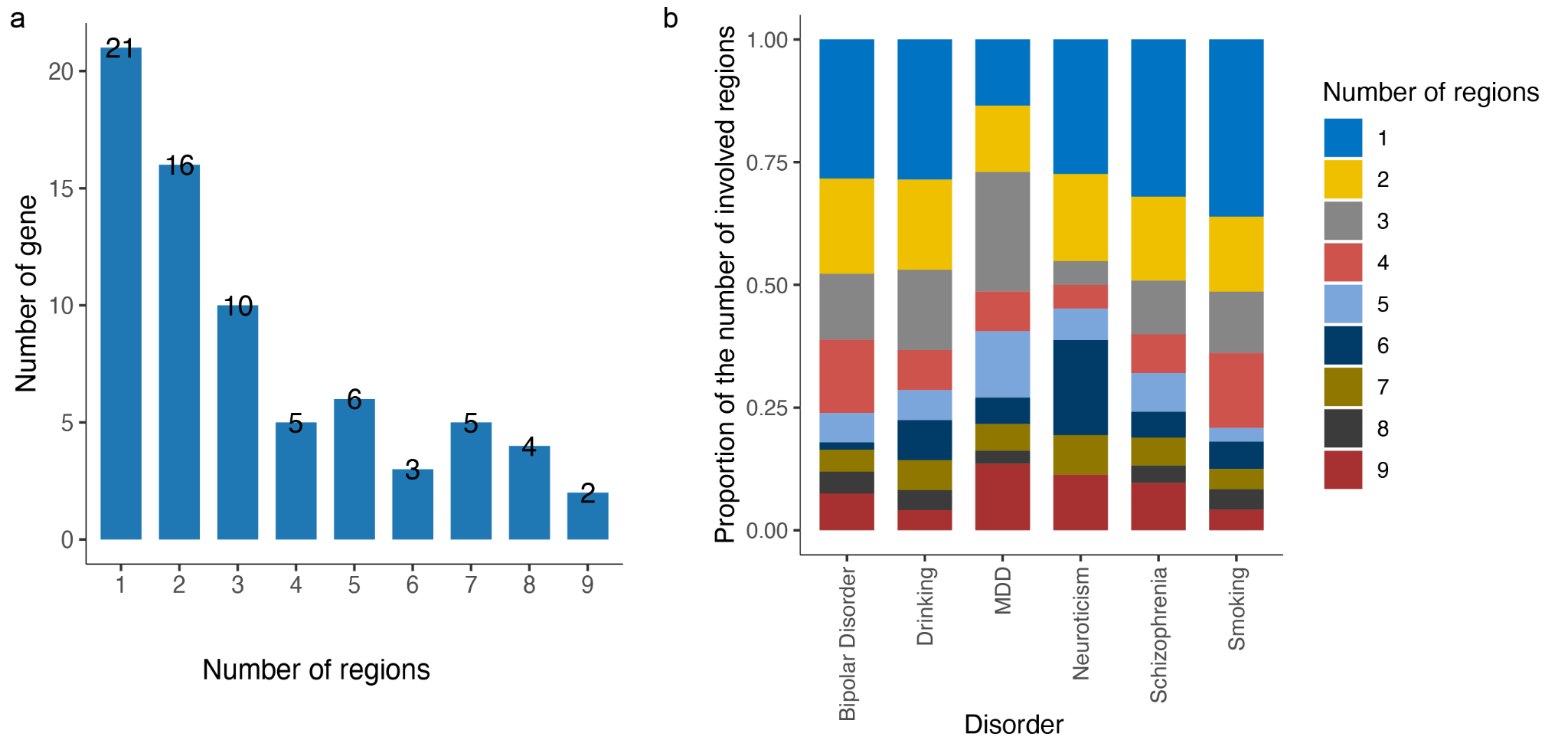


###### Supplementary Fig. **19|** **Brain region-specificity of the prioritized genes.** **a,** The number of genes that were predicted in one or multiple brain regions for SCZ-fine mapped SNPs. **b.** The distribution of the number of involved brain regions of prioritized neuropsychiatric genes for GWAS significant SNPs. MDD: Major Depressive Disorder. Source data is provided as a Source Data file.

#

#
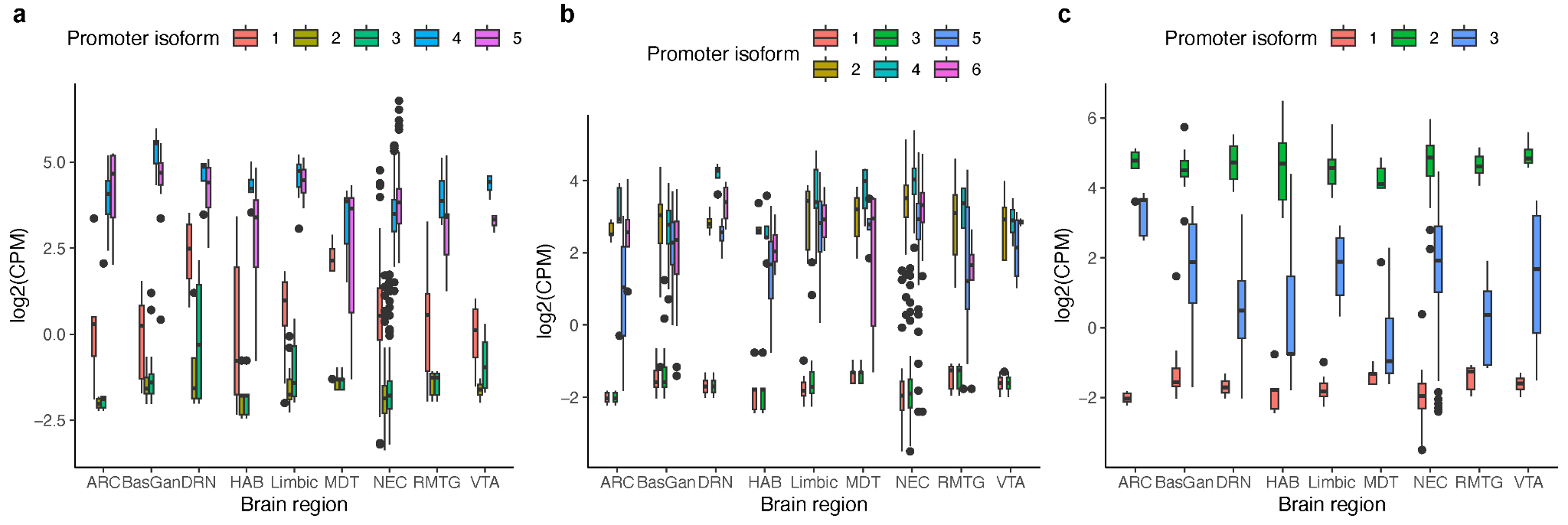


###### Supplementary Fig. **20| Expression of** promoter-isoform**s of *CALN1* (a), *FURIN* (b), and *WDR82* (c) across brain regions.** Promoter-isoforms were numerically labeled in order from the 5' to the 3' sequence. The normalized expression value and number of samples for each brain region can be found in the source data. Source data is provided as a Source Data file.

#
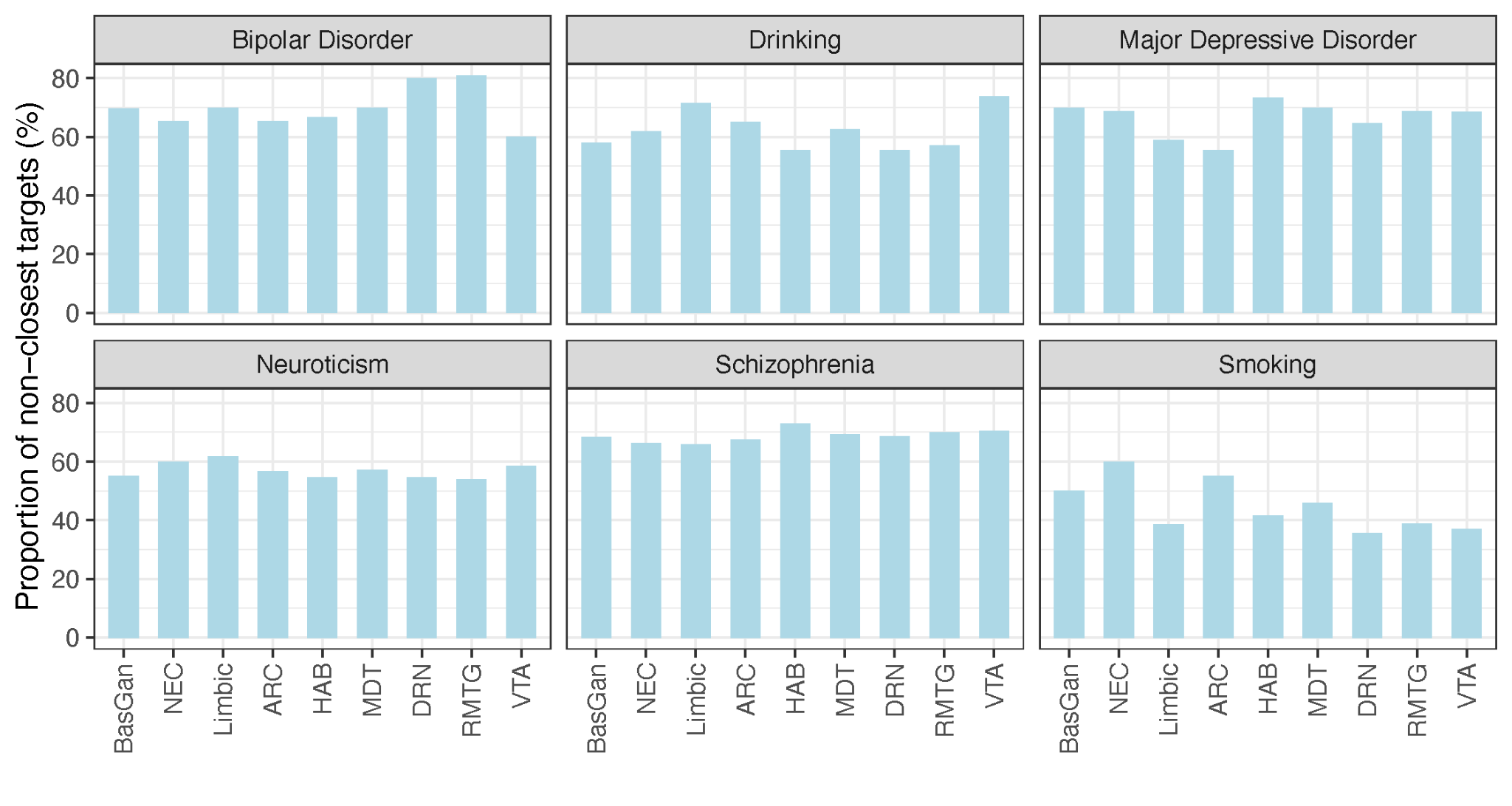


###### Supplementary Fig. 21| The percentage of genes that are not closest to lead GWAS SNP among the prioritized genes. Source data is provided as a Source Data file.

#
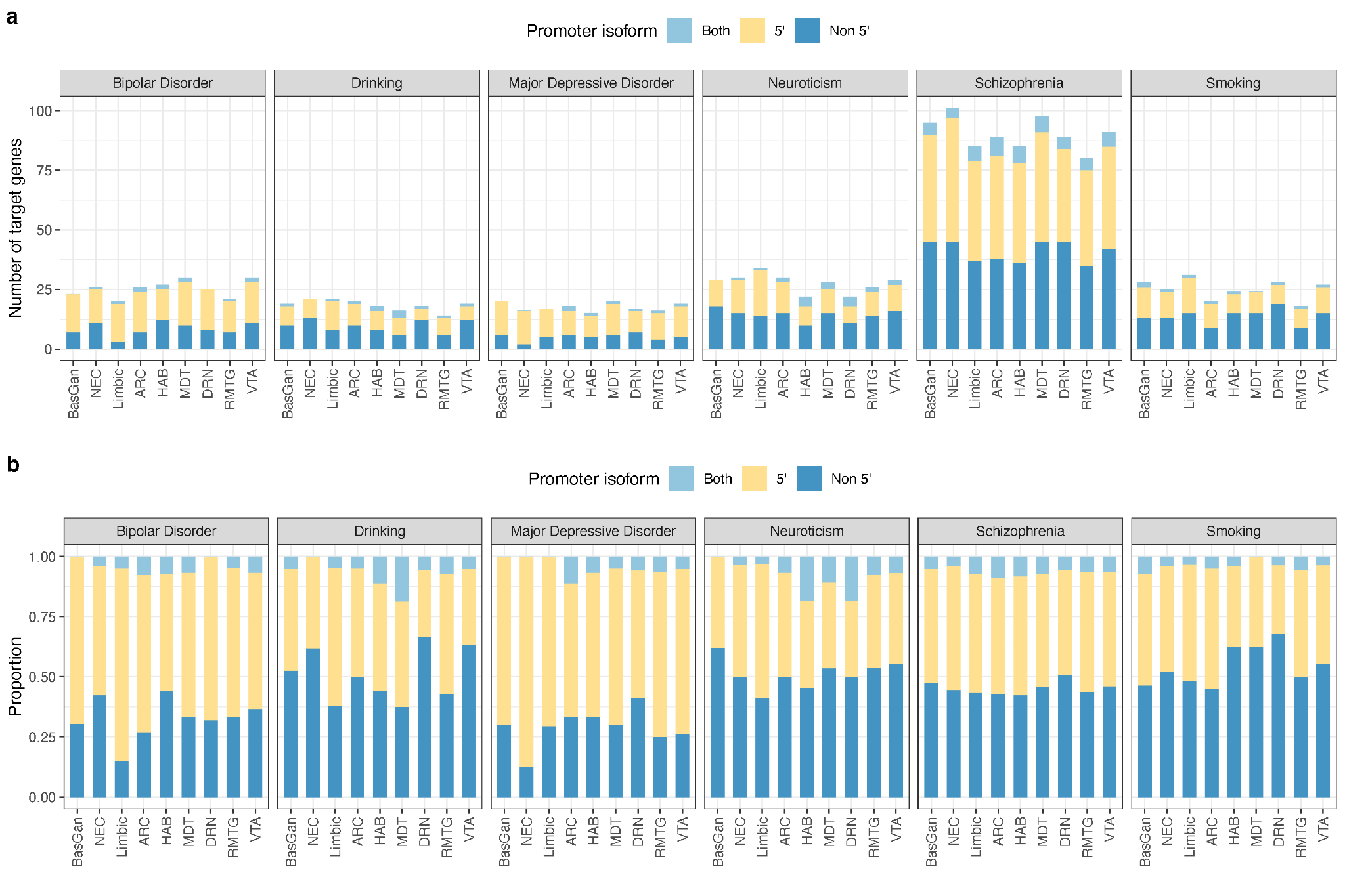


#

###### Supplementary Fig. 22| The number (a) and proportion (b) of genes identified by 5’ and non-5’ promoter-isoforms across brain regions. MDD (Major depressive disorder). Source data is provided as a Source Data file.


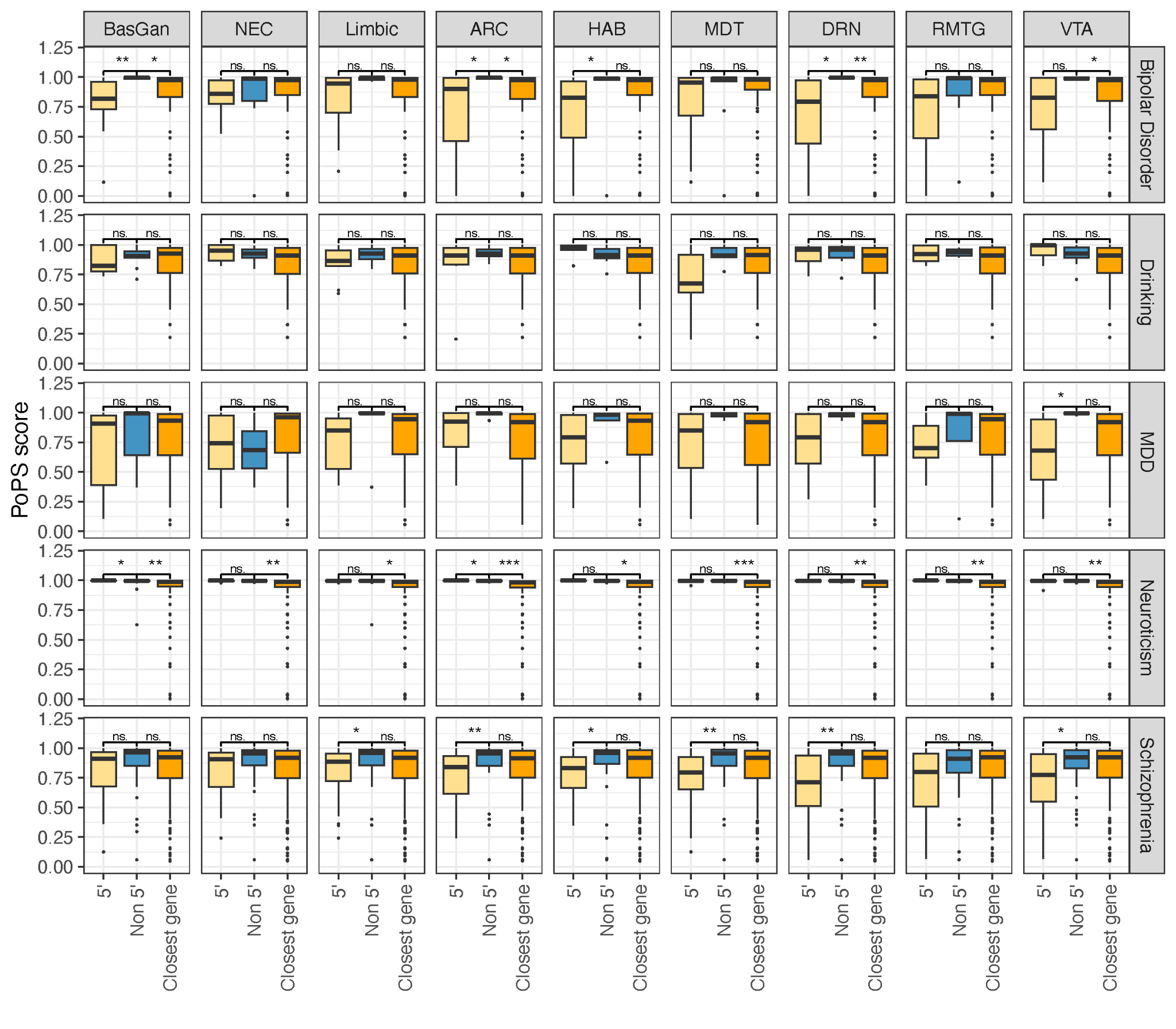


###### Supplementary Fig. **23| The distribution of PoPS score across 5’ and non-5’ targets of significant GWAS SNPs for neuropsychiatric disorders, and their closest genes.** The significance was determined with the two-tailed Wilcoxon signed-rank test. ns., *P* > 0.05; *, *P* < 0.05; **, *P* < 0.01, ***, *P* < 0.001. The PoPS score for each gene and the number of genes for each set can be found in the source data. Source data is provided as a Source Data file.

######
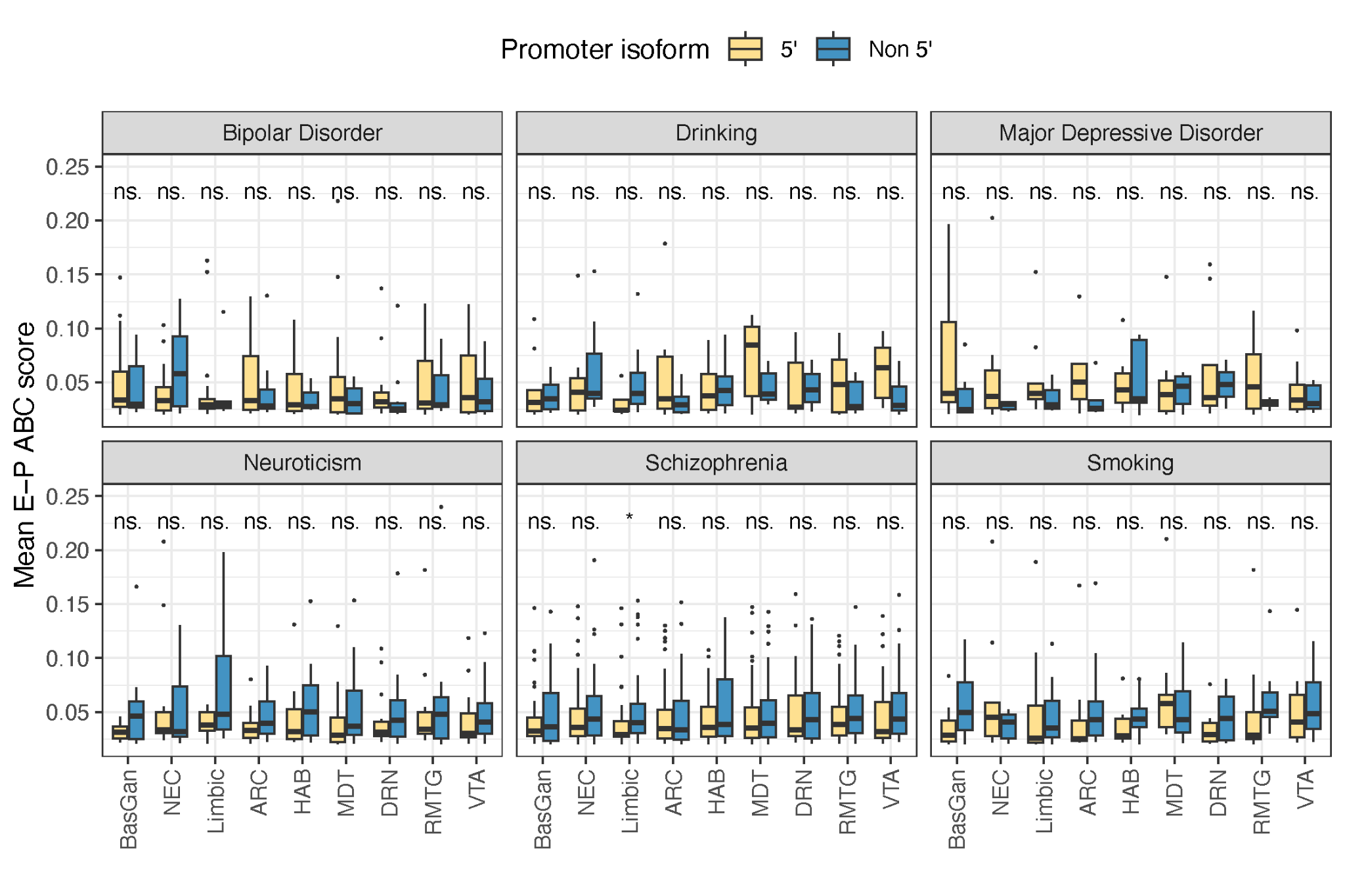


**Supplementary Fig. 24| The distribution of ABC scores of 5’ and non-5’ targets across brain regions for psychiatric disorders.** The significance of differences between 5’ and non-5’ promoter-isoforms was determined with the two-tailed Wilcoxon signed-rank test. ns., p > 0.05; *, p < 0.05. The ABC scores and the number of links for each set can be found in the source data. Source data is provided as a Source Data file.

######


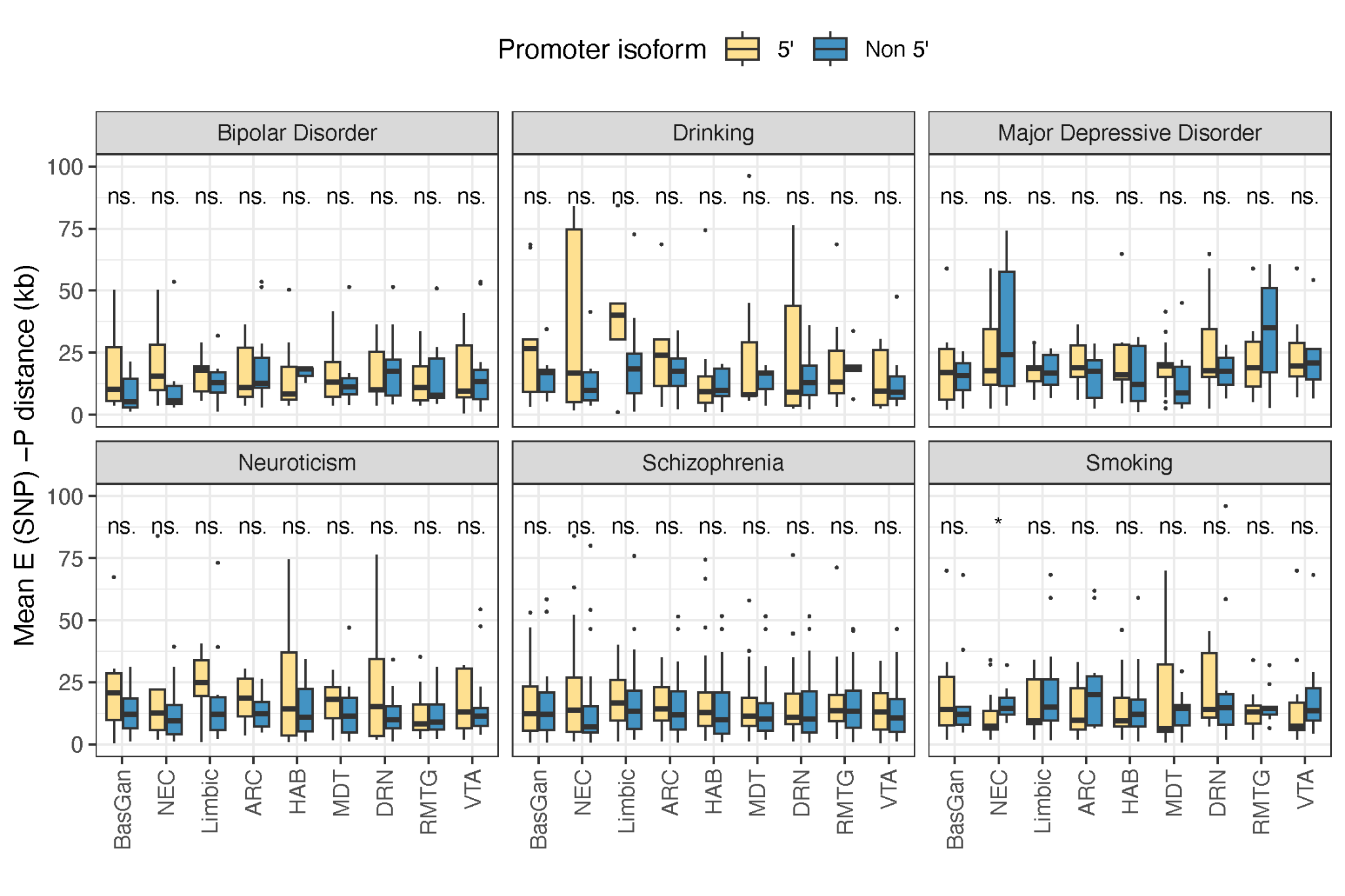


###### Supplementary Fig. **25| The distribution of distances between SNP (enhancer)** **to their target isoforms (promoter) across brain regions for psychiatric disorders.** The significance of differences between 5’ and non-5’ promoter-isoforms was determined with the two-tailed Wilcoxon signed-rank test. ns., p > 0.05; *, p < 0.05. The E-P distance and the number of links for each set can be found in the source data. Source data is provided as a Source Data file.

## Supplementary Tables

| **Subject ID** | **Brain bank** | **Age of death**  **(years)** | **Gender** | **Ancestry** | **PMI**  **(minutes)** |
| --- | --- | --- | --- | --- | --- |
| BB1 | Mount Sinai Brain Bank (MSBB) | 20 | Female | European | 1247 |
| BB2 | Mount Sinai Brain Bank (MSBB) | 35 | Male | Hispanic | 926 |
| BB3 | University of Miami, Brain Endowment Bank (BEB) | 20 | Male | European | 1680 |
| BB4 | University of Miami, Brain Endowment Bank (BEB) | 30 | Female | Afri-american | 924 |
| BB5 | University of Miami, Brain Endowment Bank (BEB) | 34 | Male | European | 1572 |
| BB6 | University of Miami, Brain Endowment Bank (BEB) | 39 | Female | European | 1302 |

### Supplementary Table 1 Demographic information of the six donors

## Reference

[1. Dong, P. *et al.* Population-level variation in enhancer expression identifies disease mechanisms in the human brain. *Nat. Genet.* **54**, 1493–1503 (2022).](https://sciwheel.com/work/bibliography/13672697)

[2. Hoffman, G. E. *et al.* CommonMind Consortium provides transcriptomic and epigenomic data for Schizophrenia and Bipolar Disorder. *Sci. Data* **6**, 180 (2019).](https://sciwheel.com/work/bibliography/8218833)

[3. Rizzardi, L. F. *et al.* Neuronal brain-region-specific DNA methylation and chromatin accessibility are associated with neuropsychiatric trait heritability. *Nat. Neurosci.* **22**, 307–316 (2019).](https://sciwheel.com/work/bibliography/6272553)

[4. Hauberg, M. E. *et al.* Common schizophrenia risk variants are enriched in open chromatin regions of human glutamatergic neurons. *Nat. Commun.* **11**, 5581 (2020).](https://sciwheel.com/work/bibliography/9969657)

[5. Fullard, J. F. *et al.* An atlas of chromatin accessibility in the adult human brain. *Genome Res.* **28**, 1243–1252 (2018).](https://sciwheel.com/work/bibliography/5486493)

[6. Corces, M. R. *et al.* Single-cell epigenomic analyses implicate candidate causal variants at inherited risk loci for Alzheimer’s and Parkinson’s diseases. *Nat. Genet.* **52**, 1158–1168 (2020).](https://sciwheel.com/work/bibliography/9909252)

[7. Yao, Z. *et al.* A taxonomy of transcriptomic cell types across the isocortex and hippocampal formation. *Cell* **184**, 3222-3241.e26 (2021).](https://sciwheel.com/work/bibliography/11050514)

[8. GTEx Consortium. The GTEx Consortium atlas of genetic regulatory effects across human tissues. *Science* **369**, 1318–1330 (2020).](https://sciwheel.com/work/bibliography/9635829)

[9. Li, Y. E. *et al.* A comparative atlas of single-cell chromatin accessibility in the human brain. *Science* **382**, eadf7044 (2023).](https://sciwheel.com/work/bibliography/15501918)

[10. Koopmans, F. *et al.* SynGO: An Evidence-Based, Expert-Curated Knowledge Base for the Synapse. *Neuron* **103**, 217-234.e4 (2019).](https://sciwheel.com/work/bibliography/7028478)
