# Supplementary material for: A multi-regional human brain atlas of chromatin accessibility and gene expression facilitates promoter-isoform resolution genetic fine-mapping
Source: Nat Commun. 2024 Nov 22;15:10113. doi: 10.1038/s41467-024-54448-y (PMC11584674; doi:10.1038/s41467-024-54448-y)
Supplement: Supplementary file 2 — Description of Additional Supplementary Files [file 41467_2024_54448_MOESM2_ESM.pdf]

Description of Additional Supplementary Files

Supplementary Data 1: Whole-genome sequencing QC metrics. Alignment and Imputation summary information for the whole genome sequencing data. Ti/Tv represent the transition/transversion ratios.

Supplementary Data 2: RNA-seq QC metrics. Quality control metrics for RNA seq during library preparation and data processing.

Supplementary Data 3: ATAC-seq QC metrics. Quality control metrics for ATAC seq during library preparation and data processing. RSC represents The relative strand correlation. NSC represents the normalized strand coefficient, PBC. PCR Bottleneck Coefficient.

Supplementary Data 4: covariates for DE analysis. The selected covariates and the description for differential gene expression, chromatin accessibility, and promoter isoform analysis.

Supplementary Data 5: Differential gene expression Significant differentially expressed genes across different brain regions. For broad brian regions, including ForeBr, BasGan, and MidDien, the statistics were derived between the given brain region and the rest of the broad brain regions. For Limbic, NEC, MidBr, and Dien, we performed pairwise comparisons between Limbic vs NEC, and MidBr vs Dien. N represents neuron, and non -N represents non -Neuron.

Supplementary Data 6: differential chromatin accessibility . Significant differentially chromatin accessibility across different brain regions. For broad brian regions, including ForeBr, BasGan, and MidDien, the statistics were derived between the given brain region and the rest of the broad brain regions. For Limbic, NEC, MidBr, and Dien, we performed pairwise comparisons between Limbic vs NEC, and MidBr vs Dien. N represents neuron, and non -N represents non -Neuron.

Supplementary Data 7: Annotated promoter -isoform . The list of annotated promoter isoforms. Entrez transcript id was assigned to corresponding promoter -isoform. If multiple transcripts share the same promoter -isoform, the id was randomly assigned to one of the transcripts.

Supplementary Data 8: Differential promoter-isoform expression. The full list of differential promoter -isoform expression analysis across brain regions in neurons.

Supplementary Data 9: alternative promoter -isoform. The list of alternative promoter - isoforms across brain regions, and the statistical test results at promoter isoform and gene level.

Supplementary Data 10: enhancer-promoter links at promoter-isoform resolution Brain region specific ABC E-P link at promoter-isoform resolution.

Supplementary Data 11: fine-mapped gene sets for neuropsychiatric traits. promoterisoform E-P link fine-mapped gene sets for SCZ, BD, and other neuropsychiatric traits.
