# Supplementary material for: A multi-regional human brain atlas of chromatin accessibility and gene expression facilitates promoter-isoform resolution genetic fine-mapping
Source: Nat Commun. 2024 Nov 22;15:10113. doi: 10.1038/s41467-024-54448-y (PMC11584674; doi:10.1038/s41467-024-54448-y)
Supplement: Supplementary file 15 — Reporting Summary [file 41467_2024_54448_MOESM15_ESM.pdf]

nature portfolio

Corresponding author(s): Pengfei Dong, Panos Roussos

Last updated by author(s): Oct 11, 2024

Reporting Summary

Nature Portfolio wishes to improve the reproducibility of the work that we publish. This form provides structure for consistency and transparency in reporting. For further information on Nature Portfolio policies, see our Editorial Policies and the Editorial Policy Checklist.

Statistics

For all statistical analyses, confirm that the following items are present in the figure legend, table legend, main text, or Methods section.

n/a	Confirmed	
☐	☒	The exact sample size (n) for each experimental group/condition, given as a discrete number and unit of measurement
☐	☒	A statement on whether measurements were taken from distinct samples or whether the same sample was measured repeatedly
☐	☒	The statistical test(s) used AND whether they are one- or two-sided<br>*Only common tests should be described solely by name; describe more complex techniques in the Methods section.*
☐	☒	A description of all covariates tested
☐	☒	A description of any assumptions or corrections, such as tests of normality and adjustment for multiple comparisons
☐	☒	A full description of the statistical parameters including central tendency (e.g. means) or other basic estimates (e.g. regression coefficient) AND variation (e.g. standard deviation) or associated estimates of uncertainty (e.g. confidence intervals)
☐	☒	For null hypothesis testing, the test statistic (e.g. F, t, r) with confidence intervals, effect sizes, degrees of freedom and P value noted<br>*Give P values as exact values whenever suitable.*
☒	☐	For Bayesian analysis, information on the choice of priors and Markov chain Monte Carlo settings
☒	☐	For hierarchical and complex designs, identification of the appropriate level for tests and full reporting of outcomes
☐	☒	Estimates of effect sizes (e.g. Cohen's d, Pearson's r), indicating how they were calculated

Our web collection on statistics for biologists contains articles on many of the points above.

Software and code

Policy information about availability of computer code

Data collection	BD FACSDiva 8.0.1
Data analysis	ABC-Enhancer-Gene-Prediction (v0.2), BEDTools (v2.25.0), bedGraphToBigwig (v4.0.0), fastqc (v0.11.2), GATK (v3.5.0), KING (v1.9), LDSC (v1.0.1), MAGMA (v1.07b), PICARD (v2.2.4), phantompeakqualtools (v2.0), plink (v1.90), qualimap (v2.0), RSEM (v1.3.1), samtools (v0.1.19), SNPRelate (v1.16.0), snpStats (v1.30.0), STAR (v2.7.2a), Trimmomatic (v0.36),<br>PoPS (v0.2), MACS2 (v2.2.7.1)<br>BEDOPS(V2.4.41)<br>ANNOVAR108 (v2020-06-08)<br>SAFFARI (https://github.com/mkoromina/SAFFARI)<br><br>R (v4.0.3) and packags<br>ChIPseeker 1.26.0, edgeR 3.32.0, GenomicFeatures 1.42.1, limma 3.46.0, Proactive 1.0.0, RSubread 1.22.0, Seurat (v4.1.1), Signac(v1.1.1.9013), variancePartition(v1.20.0), ComplexHeatmap (v2.6.2)<br>python (v3.8.15) and packages<br>Scanpy (v1.9.3, Pegasus(v1.7.1), cnmf (v1.4.1)<br>All software are freely available.

For manuscripts utilizing custom algorithms or software that are central to the research but not yet described in published literature, software must be made available to editors and reviewers. We strongly encourage code deposition in a community repository (e.g. GitHub). See the Nature Portfolio guidelines for submitting code & software for further information.

Data

Policy information about availability of data

All manuscripts must include a data availability statement. This statement should provide the following information, where applicable:

- Accession codes, unique identifiers, or web links for publicly available datasets
- A description of any restrictions on data availability
- For clinical datasets or third party data, please ensure that the statement adheres to our policy

The raw data generated in the current study are available through the Gene Expression Omnibus (GEO) under accession number GSE211826 (ATAC-seq and RNA-seq), and Sequence Read Archive (SRA) under accession number PRJNA870417 (WGS. The UCSC genome browser tracks of our processed ATAC-seq data, download links, and the updated PoPS score feature and PoPS score for different traits are available at our webpage at synapse (synID:syn35856920)
The following publicly available datasets were used: The Corces et al. human multi-brain-region single-cell ATAC-seq reference18 is available on NCBI GEO (GSE147672), the Skene et al. mouse multi-brain-region single-cell RNA-seq reference20 is available from http://www.hjerling-leffler-lab.org/data/scz_singlecell/, the Lake et al. human brain single-cell RNA-seq reference117 is available on NCBI GEO (GSE97942), GTEx human multi-brain region RNA-seq42 data is available on from https://gtexportal.org/home/, the Fullard et al. human multi-brain region ATAC-seq data8 is available on NCBI GEO (GSE96949). Source data are provided with this paper.

Research involving human participants, their data, or biological material

Policy information about studies with human participants or human data. See also policy information about sex, gender (identity/presentation), and sexual orientation and race, ethnicity and racism.

Reporting on sex and gender	Our samples come from six postmortem brains, including 3 males and 3 females. Sex was determined by self-report, and confirmed with genotype check. To check the sex of individuals within our cohort, we also measured the number of reads mapped on genes located on chromosome Y with WGS data(genes located on pseudoautosomal regions are not counted; Supplementary Fig. 2b). To check the identity of RNA-seq samples, we ran genotype comparisons of all samples against each other and against imputed genotypes from WGS.
Reporting on race, ethnicity, or other socially relevant groupings	Our samples come from six donors, including 4 European ancestries, 1 Hispanic, and 1 African American. To verify ancestry information, we merged the whole genome samples with 1KG cohort and performed principal component analysis on the thinned set of 30,000 randomly selected SNPs with MAF ≥ 5% (SNPRelate package v1.16). Based on the proximity of our samples to 1KG population clusters in the two-dimensional space of the first two principal components, we checked the ethnicity information of all six individuals (Supplementary Fig. 2a).
Population characteristics	The details of the population structure can be found supplementary table 1
Recruitment	Four subjects (2x male, 2x female) were collected at autopsy at the Brain Endowment Bank (BEB) at Miller School of Medicine at the University of Miami, and 2 subjects (1x male, 1x female) were collected from Mount Sinai Brain Bank (MSBB).<br>All brain specimens were obtained through informed consent and/or brain donation programs at the Miller School of Medicine at the University of Miami and the Icahn School of Medicine at Mount Sinai.
Ethics oversight	All procedures and research protocols were approved by Institutional Review Boards from Miller School of Medicine at the University of Miami and the Icahn School of Medicine at Mount Sinai.

Note that full information on the approval of the study protocol must also be provided in the manuscript.

Field-specific reporting

Please select the one below that is the best fit for your research. If you are not sure, read the appropriate sections before making your selection.

☒ Life sciences ☐ Behavioural & social sciences ☐ Ecological, evolutionary & environmental sciences

For a reference copy of the document with all sections, see nature.com/documents/nr-reporting-summary-flat.pdf

Life sciences study design

All studies must disclose on these points even when the disclosure is negative.

Sample size	Human brain samples: 265 RNA-seq and 202 ATAC-seq after sample exclusion.
Data exclusions	RNA-seq: 17 samples had a technical or biological replicate. We decided to keep those replicates with a better correlation to the gene expression profiles of the other samples originating from the same cell type and brain region. 26 samples were removed after an inspection of metadata which identified probable reasons such as the low amount of starting material, low RIN, or low ratio of uniquely mapped reads.<br>ATAC-seq: 83 libraries did not pass QC thresholds as described in the Methods, i.e., either low GC content, low mappability, low number of sequencing reads, and/or low fraction of reads in peaks.<br>Additionally, we inspected all ATAC-seq libraries in IGV browser and removed an additional 8 samples with the lowest TSS enrichment and/or less than 1,000 nuclei.

Replication	(1) We have successfully validated the cell-type and brain-region specificity of our RNA-seq and ATAC-seq with single-cell references.<br>(2) We have successfully validated our promoter-isoform expression with promoter OCR activity across brain regions.<br>(3) We have used published, independent QTL data sets successfully validated our enhancer-promoter links at promoter isoform resolution.<br>(4) We have used indepdent annotation from PoPS to succesffully validate our promoter-isoform level genetic fine mapping.
Randomization	Generation of RNA-seq and ATAC-seq libraries were randomized for brain region, cell type at multiple steps including:<br>1. During FANS and/or transposase reaction<br>2. PCR amplification for library preparation/barcode assignment<br>3. Gel extraction<br>4. Sequencing pools
Blinding	Data were blinded to the outcome measures.

Reporting for specific materials, systems and methods

We require information from authors about some types of materials, experimental systems and methods used in many studies. Here, indicate whether each material, system or method listed is relevant to your study. If you are not sure if a list item applies to your research, read the appropriate section before selecting a response.

Materials & experimental systems

n/a	Involved in the study	
☐	☒	Antibodies
☒	☐	Eukaryotic cell lines
☒	☐	Palaeontology and archaeology
☒	☐	Animals and other organisms
☒	☐	Clinical data
☒	☐	Dual use research of concern
☒	☐	Plants

Methods

n/a	Involved in the study	
☒	☐	ChIP-seq
☐	☒	Flow cytometry
☒	☐	MRI-based neuroimaging

Antibodies

Antibodies used	FANS-sorting of brain tissue: Anti-NeuN Antibody, clone A60, Alexa Fluor488 conjugated<br>(1:1000, Millipore, Cat# MAB377X)
Validation	Antibody has been validated by the respective manufacturerand was also validated by many publications that used this antibody as cited on manufacturer's website.<br>https://www.emdmillipore.com/US/en/product/Anti-NeuN-Antibody-clone-A60-Alexa-Fluor488-conjugated,MM_NF-MAB377X#documentation

Plants

Seed stocks	*Report on the source of all seed stocks or other plant material used. If applicable, state the seed stock centre and catalogue number. If plant specimens were collected from the field, describe the collection location, date and sampling procedures.*
Novel plant genotypes	*Describe the methods by which all novel plant genotypes were produced. This includes those generated by transgenic approaches, gene editing, chemical/radiation-based mutagenesis and hybridization. For transgenic lines, describe the transformation method, the number of independent lines analyzed and the generation upon which experiments were performed. For gene-edited lines, describe the editor used, the endogenous sequence targeted for editing, the targeting guide RNA sequence (if applicable) and how the editor was applied.*
Authentication	*Describe any authentication procedures for each seed stock used or novel genotype generated. Describe any experiments used to assess the effect of a mutation and, where applicable, how potential secondary effects (e.g. second site T-DNA insertions, mosiacism, off-target gene editing) were examined.*

Flow Cytometry

Plots

Confirm that:

☒ The axis labels state the marker and fluorochrome used (e.g. CD4-FITC).

☒ The axis scales are clearly visible. Include numbers along axes only for bottom left plot of group (a 'group' is an analysis of identical markers).

☒ All plots are contour plots with outliers or pseudocolor plots.

☒ A numerical value for number of cells or percentage (with statistics) is provided.

Methodology

Sample preparation	50 mg of frozen brain tissue was homogenized in chilled lysis buffer (0.32 M Sucrose, 5 mM CaCl2, 3 mM Magnesium acetate, 0.1 mM, EDTA, 10 mM Tris-HCl, pH 8, 1 mM DTT, 0.1% Triton X-100) and filtered through a 40 µm cell strainer. Filtered lysate was underlaid with sucrose solution (1.8 M Sucrose, 3 mM Mg(CH3COO)2, 1 mM DTT, 10 mM Tris-HCl, pH 8) and subjected to ultracentrifugation at 107,000 xg for 1 hour at 4 ℃. Pellets were resuspended in 500 µl DPBS containing 0.1% BSA. anti-NeuN antibody (1:1000, Alexa488 conjugated, Millipore, Cat# MAB377X) was added and samples incubated, in the dark, for 1 hr at 4 ℃. Prior to FANS sorting, DAPI (Thermoscientific) was added to a final concentration of 1 µg/ml. DAPI positive neuronal (NeuN+) and non-neuronal (NeuN-) nuclei were isolated using a FACSAria flow cytometer with FACSDiva Version 8.0.1 software (BD Biosciences).
Instrument	FACSAria flow cytometer (BD Biosciences)
Software	FACSDiva software, version 8 (BD Biosciences)
Cell population abundance	Across all samples, the average abundance of each nuclei type:<br>NeuN+: 27.5%<br>NeuN-: 72.5%<br>Following FANS, sample concentrations were cofirmed using a Countess II Cell Counter (Life Technologies)
Gating strategy	Nuclear populations were initially gated to side and forward scatter to initiate nuclei from cellular debris. Populations were then gated based on DAPI staining to identify singlets and to further remove debris. DAPI positive nuclei were subsequently gated based on NeuN staining to differentiate neurons from non-neurons.

☒ Tick this box to confirm that a figure exemplifying the gating strategy is provided in the Supplementary Information.
